# Integrative omics-analysis of lipid metabolism regulation by peroxisome proliferator-activated receptor a and b agonists in male Atlantic cod

**DOI:** 10.3389/fphys.2023.1129089

**Published:** 2023-03-22

**Authors:** Marta Eide, Anders Goksøyr, Fekadu Yadetie, Alejandra Gilabert, Zdenka Bartosova, Håvard G. Frøysa, Shirin Fallahi, Xiaokang Zhang, Nello Blaser, Inge Jonassen, Per Bruheim, Guttorm Alendal, Morten Brun, Cinta Porte, Odd André Karlsen

**Affiliations:** ^1^ Department of Biological Sciences, University of Bergen, Bergen, Norway; ^2^ Institute of Environmental Assessment and Water Research, Spanish National Research Council (CSIC), Barcelona, Spain; ^3^ Faculty of Science, National Distance Education University (UNED), Madrid, Spain; ^4^ Department of Biotechnology and Food Science, Norwegian University of Science and Technology (NTNU), Trondheim, Norway; ^5^ Department of Mathematics, University of Bergen, Bergen, Norway; ^6^ Institute of Marine Research (IMR), Bergen, Norway; ^7^ Computational Biology Unit, Department of Informatics, University of Bergen, Bergen, Norway; ^8^ Faculty of Medicine, University of Oslo, Oslo, Norway; ^9^ Department of Informatics, University of Bergen, Bergen, Norway

**Keywords:** fish, lipidomics, transcriptomics, proteomics, WY-14,643, GW501516, PPAR

## Abstract

Lipid metabolism is essential in maintaining energy homeostasis in multicellular organisms. In vertebrates, the peroxisome proliferator-activated receptors (PPARs, NR1C) regulate the expression of many genes involved in these processes. Atlantic cod (*Gadus morhua*) is an important fish species in the North Atlantic ecosystem and in human nutrition, with a highly fatty liver. Here we study the involvement of Atlantic cod Ppar a and b subtypes in systemic regulation of lipid metabolism using two model agonists after *in vivo* exposure. WY-14,643, a specific PPARA ligand in mammals, activated cod Ppara1 and Ppara2 *in vitro*. *In vivo*, WY-14,643 caused a shift in lipid transport both at transcriptional and translational level in cod. However, WY-14,643 induced fewer genes in the fatty acid beta-oxidation pathway compared to that observed in rodents. Although GW501516 serves as a specific PPARB/D ligand in mammals, this compound activated cod Ppara1 and Ppara2 as well as Pparb *in vitro*. *In vivo*, it further induced transcription of Ppar target genes and caused changes in lipid composition of liver and plasma. The integrative approach provide a foundation for understanding how Ppars are engaged in regulating lipid metabolism in Atlantic cod physiology. We have shown that WY-14,643 and GW501516 activate Atlantic cod Ppara and Pparb, affect genes in lipid metabolism pathways, and induce changes in the lipid composition in plasma and liver microsomal membranes. Particularly, the combined transcriptomic, proteomics and lipidomics analyses revealed that effects of WY-14,643 on lipid metabolism are similar to what is known in mammalian studies, suggesting conservation of Ppara functions in mediating lipid metabolic processes in fish. The alterations in the lipid profiles observed after Ppar agonist exposure suggest that other chemicals with similar Ppar receptor affinities may cause disturbances in the lipid regulation of fish. Model organism: Atlantic cod (*Gadus morhua*). LSID: urn:lsid:zoobank.org:act:389BE401-2718-4CF2-BBAE-2E13A97A5E7B. COL Identifier: 6K72F.

## 1 Introduction

The degradation of dietary and stored fat and biosynthesis of structural and functional lipids, commonly known as lipid metabolism, is essential in maintaining energy homeostasis in multicellular organisms. In vertebrates, a group of nuclear receptor transcription factors named peroxisome proliferator-activated receptors (PPARs, NR1C) regulate the expression of many genes involved in these processes (Schoonjans et al., 1996). Three related subtypes with distinct tissue distribution, ligand specificities, and target gene repertoire have been identified in vertebrates (Chinetti et al., 2000; Grygiel-Gorniak, 2014). PPARalpha (PPARA, *NR1C1*) is mainly expressed in tissues with high catabolic rates, such as the liver, heart, and muscle, and is a key regulator of fatty acid β-oxidation. PPARbeta/delta (PPARB/D, *NR1C2*) is another regulator of the fatty acid metabolism that, in comparison to PPARA, is more ubiquitously expressed in different tissues, including in adipose tissue. Lastly, the PPARgamma (PPARG, *NR1C3*) subtype is mainly expressed in white and brown adipose tissues and is the major regulator of adipocyte differentiation and maturation (Lehrke and Lazar, 2005).

A wide variety of synthetic and natural ligands have been shown to activate PPARs. In mammals, long-chain mono- and polyunsaturated fatty acids (FA), such as linoleic acid (18:2n-6), arachidonic acid (20:4n-6), and their derivatives are established endogenous peroxisome proliferators and PPAR agonists (Desvergne and Wahli, 1999; Hihi et al., 2002). Synthetic PPAR ligands include lipid-lowering pharmaceuticals such as fibrates, and environmental contaminants, such as phthalate mono-esters and perfluorinated compounds (Bility et al., 2004; White et al., 2011). The potential metabolic-disrupting effects of these chemicals on lipid metabolism have accentuated their putative involvement in the human epidemic of obesity and obesity-related metabolic disorders (Grün and Blumberg, 2009; Casals-Casas and Desvergne, 2011).

Ppar subtypes have been cloned and studied in several teleost fish species. These include plaice (*Pleuronectes platessa,* Pleuronectidae), gilthead seabream (*Sparus aurata*), loath (*Misgurnus anguillicaudatus*, Cobitidae), brown trout (*Salmo trutta f. fario,* Salmonidae), Atlantic salmon (*Salmo salar,* Salmonidae), sea bream (*S. aurata*, Sparidae), and sea bass (*Dicentrarchus labrax,* Moronidae) (Ruyter et al., 1997; Andersen et al., 2000; Boukouvala et al., 2004; Leaver et al., 2005; Leaver et al., 2007; Liang et al., 2016; Madureira et al., 2019). For many of them, multiple homologs are present for each Ppar subtype due to the teleost-specific genome duplications (Robinson-Rechavi et al., 2001; Meyer and Van de Peer, 2005). Still, the tissue distribution, ligand activation, and physiological role of fish Ppara and Pparb seem to be similar to what is found in mammalian species. However, in contrast to Ppara and Pparb, teleost Pparg is either not or only weakly activated by classical mammalian PPARG agonists (e.g., rosiglitazone), most likely due to large divergence of the amino acid sequence constituting the ligand-binding domains (Andersen et al., 2000; Leaver et al., 2005; Capitao et al., 2018).

Atlantic cod (*Gadus morhua,* Gadidae) is one of the most important teleost species in North Atlantic fisheries and an important nutritional source for humans. The cod liver contains >50% total fat, which is mostly stored as triglycerides in intracellular lipid droplets (Jangaard et al., 1967). Moreover, half of the fatty acids present in cod muscle are eicosapentaenoic acid (EPA 20:5n-3) and docosahexaenoic acid (DHA 22:6n-3), also known as essential omega-3 fatty acids with well-described health benefits (Zhang et al., 2019). Due to its widespread habitat and key role in North-Atlantic marine ecosystems, the Atlantic cod has commonly been included in environmental monitoring programs and is increasingly used as a model species in toxicological studies (Meier et al., 2010; Yadetie et al., 2013; Eide et al., 2014; Bizarro et al., 2016; Yadetie et al., 2018; Dale et al., 2019; Khan et al., 2019; Olsvik et al., 2019). Furthermore, the availability of a sequenced and annotated genome (Star et al., 2011) together with the toxicogenomic and bioinformatics approaches, has revealed evolved differences in both the immune- and the xenobiotic response systems (Malmstrøm et al., 2013; Eide et al., 2018). Recent studies on Atlantic cod have demonstrated that certain environmental pollutants affect lipid levels and composition, including perfluoroalkylated substances (PFAS) (Dale et al., 2020; Dale et al., 2021) and the non-dioxin-like polychlorinated biphenyl PCB153 (Yadetie et al., 2014). Specifically, hepatic proteome analysis identified that pathways related to lipid degradation pathways, including fatty acid β-oxidation, were significantly affected by PFAS exposure (Dale et al., 2020). We have recently cloned the four Ppars found in the Atlantic cod genome, Ppara1 and Ppara2, Pparb, and Pparg, and studied their tissue-specific expression and ligand activation characteristics with mammalian model-ligands and a selected set of PFAS (Søderstrøm et al., 2022). However, the activation of cod Ppars *in vivo* and their regulatory role in lipid metabolism is not well understood or described.

The objective of this study was to investigate the involvement of Ppar activation in the systemic regulation of lipid metabolism in Atlantic cod using two model PPAR agonists, WY-14,643 (pirinixic acid) and GW501516 (cardarine), which in humans and rodents are known to specifically activate PPARA and PPARB, respectively. Following *in vivo* injections, we studied the responses at the transcriptomic and proteomic levels in liver, and at the metabolomic (lipidomic) level in liver and plasma, using state-of-the-art omics technologies. Applying multivariate statistical and bioinformatic analyses on these multi-omics datasets we set out to interlink processes related to lipid metabolism at transcriptional, translational, and metabolomic levels in Atlantic cod, a species with a highly specialized lipid storage system.

## 2 Materials and methods

### 2.1 Luciferase reporter gene assay

Luciferase reporter gene assays were conducted essentially as described previously by (Lille-Langøy et al., 2015; Søderstrøm et al., 2022). In short, COS-7 simian kidney cells were seeded to 96 well plates (10,000 cells per well) in Dulbecco’s modified Eagle medium (DMEM) with phenol red, supplemented with 10% fetal bovine serum (FBS), 4 mM L-glutamate, 1 mM sodium pyruvate and 100 U/mL penicillin-streptomycin (Merck, KGaA, Darmstadt, Germany) and cultivated at 37°C with 5% CO_2_ for 24 h. Cells were transiently co-transfected with a eukaryotic expression plasmid (pCMX-GAL4-gmPPAR_x_) encoding the hinge and ligand-binding domain of either cod Ppara1 (gmPpara1), gmPpara2, or gmPparb fused to the GAL4 DNA-binding domain (4.76 ng/well), the tk (MH100)x4 luciferase reporter plasmid (47.6 ng/well) and the β-galactosidase normalization plasmid (pCMV-βGAL, 47.6 ng/well), using Mirus TransIT LT-1 transfection reagent according to the recommendations of the supplier. Following transfection, cells were exposed to WY-14,643 (4-Chloro-6-(2,3-xylidino)-2-pyrimidinylthioacetic acid, Pirinixic acid; Sigma Aldrich), GW501516 (2-[2-Methyl-4-[[[4-methyl-2-[4-(trifluoromethyl)phenyl]-5-thiazolyl]methyl]thio]phenoxy]-acetic acid; Sigma Aldrich), or to solvent control (dimethyl sulfoxide, DMSO; Sigma Aldrich) dissolved in DMEM supplemented with 10% charcoal-stripped FBS (VWR International, Radnor, United States) at increasing concentrations for 24 h. Reporter gene assays were repeated at least three times and with three technical replicates per exposure. Concentration-response curves were calculated by non-linear regression (4 parameters, GraphPad PRISM v. 9).

### 2.2 Atlantic cod

Atlantic cod were obtained from Havbruksstasjonen in Tromsø AS (Nofima, Tromsø, Norway) and maintained at the Industrial and Aquatic Laboratory (ILAB, Bergen, Norway). Juvenile Atlantic cod approximately 15 months old were kept in 500 L tanks in 6°C, 34 ppt seawater with a 12 h light/12 h dark cycle. The fish were fed *ad lib* with a commercial diet (Amber Neptune, batch no. 3343368, Skretting, Stavanger, Norway) until acclimatization, when feeding was restrained to 1% of the biomass daily. For feed composition and ingredients, see Supplementary Table S1. The mean body weight ±standard deviation of the fish at the start of the experiment was 205 ± 37 g. The reproductive immaturity of the fish was confirmed at sampling. The fish were maintained and treated in accordance with the guidelines of the Norwegian Board of Biological Experiments with Living Animals and with the necessary approval (FOTS ID 11730).

### 2.3 *In vivo* experimental design

WY-14,643 and GW501516 were dissolved in 10% DMSO, 90% teleost saline (2.41 mM KCl, 133.5 mM NaCl, 1.5 mM CaCl_2_, 0.79 mM MgSO_4_, 1 mM NaHCO_3_, 0.5 mM Na_2_HPO_4_). The final concentration administered were 4.0 (low dose) and 40 mg/kg (high dose) for WY-14,643, and 0.4 (low dose) and 4.0 mg/kg (high dose) for GW501516.

The fish were acclimatized in 150 L treatment tanks for 16 days prior to exposure and fed daily 1% of their biomass. In total, 109 fish were divided into five different groups, with 21–22 individuals in each group: Solvent control (Control), low and high dose WY-14,643 (WY_LD and WY_HD), and low and high dose GW501516 (GW_LD and GW_HD). Four fish belonging to different treatment groups died or were euthanized before sampling. The fish were injected intraperitoneally on day 0 and day 4 and sampled on day 11.

At sampling, the fish were killed with a blow to the head, and weight, length, and sex were registered (see section Data Availability Statement). Samples of blood, liver, bile, muscle, head kidney, and gills were collected, snap-frozen in liquid nitrogen, and stored at −80°C. Only samples from male fish (*n* = 51) were chosen for the subsequent analyses presented here.

### 2.4 Transcriptomics

#### 2.4.1 RNA extraction

Total RNA was isolated from liver tissue of 50 male fish (*n* = 9–12 per group). RNA was isolated from 50 mg of each liver tissue using TRI Reagent (Sigma, Catalog Number T9424) according to the manufacturer’s protocol. The concentration of total RNA was measured using a NanoDrop ND-1000 spectrophotometer (NanoDrop Technologies, Wilmington, DE, United States, RRID:SCR_016517), and RNA quality was assessed using the Agilent 2100 Bioanalyzer (Agilent Technologies, Palo Alto, CA).

#### 2.4.2 RNA-seq

RNA sequencing was performed at the Genomics Core Facility at the University of Bergen as previously described by (Yadetie et al., 2018). Briefly, each RNA sample (0.4 μg) was processed and sequenced using Illumina^®^ TruSeq^®^ Stranded mRNA Sample Preparation Kits according to the Illumina TruSeq^®^ Stranded mRNA Sample Preparation Guide on Illumina HiSeq 4000 (Illumina, Inc, San Diego, CA, United States, RRID:SCR_016386). Poly(A)+ RNA was purified, fragmented, and converted to first strand and second cDNAs. The second strand cDNA was amplified using PCR (15 cycles) to create the final cDNA library, which was sequenced to generate approximately 50 million 75 bp paired-end reads per sample.

#### 2.4.3 RNA-seq analysis

The RNA-seq data were processed and analyzed using the RASflow workflow pipeline (Zhang and Jonassen, 2020). The quality of the FASTQ files was checked by FastQC v0.11.8 (https://www.bioinformatics.babraham.ac.uk/projects/fastqc/, RRID:SCR_014583). HISAT2 v2.1.0 (Kim et al., 2019) and featureCounts (from subread v1.6.4) (Liao et al., 2014) were then used to quantify the gene expression level by mapping the sequencing reads to gadMor1 (Star et al., 2011). Differential gene expression analysis was then performed using edgeR v3.26.0 (RRID:SCR_012802) (Robinson et al., 2010) to produce the lists of differentially expressed genes (DEGs, FDR<0.05) used for further analysis. Pathway and network enrichment analysis was performed based on human orthologs of Atlantic cod genes retrieved from Ensembl database as described previously (Yadetie et al., 2018).

### 2.5 Proteomics

#### 2.5.1 Protein isolation

Proteomics were performed on liver tissue from 51 male fish (*n* = 9–12 per group) from this exposure study. Proteomics analyses were performed at the Proteomics Unit at the University of Bergen (PROBE). Sample preparation was performed as described previously (Yadetie et al., 2016): 20–100 mg of cod liver tissue was homogenized by sonication in a lysis buffer consisting of 8 M UREA, 0.1 M Tris-HCl, pH 8.5, 20 mM methylamine (10 µL added per mg tissue). After homogenization, samples were incubated at 37°C for 5 min, centrifuged at 13,000 rpm for 10 min, before protein concentrations in the lysates (supernatants) were determined with the Pierce BCA-protein assay kit (Thermo Fischer Scientific, Waltham, MA, United States). 30 μg of protein from each sample was transferred to an LB Eppendorf tube, and volumes adjusted to 20 µL. 20 µL of 50 mM Tris-HCl (pH 7.9) was added, and samples were incubated at room temperature (RT) for 5 min. Reduction and subsequent alkylation of the samples were performed by addition of 4 µL 100 mM DTT and 5 µL of iodoacetamide, respectively, with 1-h incubation at RT for each reaction. 0.8 mL 100 mM DTT was thereafter added to quench unreacted iodoacetamide and avoid unwanted protease alkylation. Protein samples were then digested with trypsin (PROMEGA, Sequencing Grade Modified Trypsin) for 16 h at 37°C by adding 110 µL 50 mM Tric-HCl, pH 7.9, and 0.6 mg trypsin. Trypsin digestion was stopped by adding 10% TFA to a 1% final concentration and resulting peptide samples were desalted with OASIS C18 filtration (Waters) and dried in a freezevac. Samples were reconstituted in 0.1 M TEAB for labelling with TMT 10-plex reagents (Thermo Fischer Scientific).

#### 2.5.2 Tandem mass tag (TMT) protein analysis

Nine identical reference samples were made by combining an aliquot of each sample following the protocol and guidelines provided by Thermo Fischer Scientific. Samples, including reference samples, were tagged by TMT 10 plex, and divided into nine pooled samples. Pooled samples were fractionated with the Pierce High pH Reversed-Phase Peptide Fractionation Kit, resulting in eight individual fractions for each TMT experiment. LC-MS/MS analysis was carried out as described in (Kroksveen et al., 2017), with an Ultimate 3000 RSLC system (Thermo Fischer Scientific) connected to a Q-Exactive HF equipped with an EASY-spray ion source (Thermo Fischer Scientific). A 2-h LC gradient was used. LC-MS data from the TMT-10 plex experiments were analysed in Proteome Discoverer 1.4 (Thermo Fischer Scientific) using Sequest and MS Amanda (version 1.4.4.2822).

Data treatment and statistical analyses were conducted as described in (Lereim et al., 2016). The Atlantic cod genome assembly ENSEMBL gadMor1 was used for protein identifications with Protein Discovery.

### 2.6 Untargeted lipidomics

Untargeted lipidomics analysis was performed on plasma and liver samples of twenty male fish randomly chosen from each group (*n* = 4 per group) at Per Bruheim’s lab at NTNU, Norway, using Liquid Chromatography Hybrid Quadrupole Mass Spectrometry (UPLC-HDMS).

#### 2.6.1 Lipid extraction

Lipids from cod plasma (50 µL) and liver tissue (50 mg) were extracted using the solvent system based on the Folch method (Folch et al., 1957). Liver tissue was homogenized with zirconium oxide beads (0.5 ± 0.01 g, φ 1.4 mm) in 500 µL of a cold mixture of chloroform/methanol (2:1, v/v) using a Precellys^®^24 bead homogenizer equipped with a Cryolys temperature controller (all Bertin Technologies SAS, Lund, Sweden) for tissue disruption. The sample was homogenized twice at 6500 rpm for 30 s with an intermediate 15 s pause. Lipids from plasma samples were extracted by shaking for 10 min (Thermoshaker Thermal Shake lite, VWR, Norway) with 1000 µL of cold chloroform/methanol (2:1, v/v). The liver homogenates were diluted with 500 µL of cold chloroform/methanol (2:1, v/v) prior the shaking step. Phase separation was induced by adding 200 µL of water. After 10 min of shaking (1500 rpm, 16°C) the tube was centrifuged for 6 min at maximum speed (13,400 rpm) using a small centrifuge (MiniSpin, Eppendorf, Hamburg, Germany). 400 μL of chloroform layer (lower) were collected and the resulting extract was filtrated through a syringe filter with GHP membrane, 0.2 µm, φ 13 mm (Acrodisc^®^, Pall Laboratory, Port Washington, NY, United States) and kept in a dark glass vial with a PTFE lined lid. Lipid extracts were stored at −20°C until further analysis. Prior to injection to the chromatographic system, the obtained lipid extracts were diluted with a mixture of isopropanol/acetonitrile/water (2:1:1, v/v/v).

#### 2.6.2 Sample analysis

A lipid profile analysis was performed using a UPLC separation system (Acquity UPLC^®^ I-class system) coupled to a hybrid quadrupole orthogonal time-of-flight mass spectrometer SYNAPT G2-S HDMS (both Waters, Milford, MA, United States). The previously described analytical method described by (Isaac et al., 2011) was adopted and modified. An Acquity UPLC^®^ CSH C18 analytical column (100 mm × 2.1 mm I.D, 1.7 mm) was equipped with an Acquity UPLC^®^ CSH C18 VanGuard™Pre-Column (5 mm × 2.1 mm I.D, 1.7 mm), both Waters, Milford, MA, United States. The mobile phase A consisted of a mixture of acetonitrile/water (60:40, v/v), while the mobile phase B consisted of a mixture of isopropanol/acetonitrile (90:10, v/v). Both eluent A and B contained 10 mM ammonium formate and 0.1% formic acid. The gradient elution program was as follows: 0 min, 40% B; 2 min, 43% B; 2.1 min, 50% B; 12 min 54% B; 12.1 min, 70% B; 18 min 99% B; 22.5 min 99% B; 22.6 min 40% B; 25 min 40% B. The column temperature was maintained at 55°C, the flow rate of the mobile phase was 0.35 mL/min and the injection volume was 5 µL.

Mass spectrometer operated in MS^E^ mode enabling automatic acquisition of MS/MS fragmentation spectra and the collision energy ramp from 15 to 35 V. Data were acquired over the mass range of 50–1999 Da. Positive ion electrospray ionization modes were applied and the MS tuning parameters were set as follows: capillary voltages 2.8 kV, the source temperature 120°C, the desolvation temperature 500°C, the cone gas flow 50 L/h, the desolvation gas flow 900 L/h, and the nebulizer gas pressure 6 bar. Leucine enkephalin was used as the lock mass.

#### 2.6.3 Data processing

Data were collected using the MassLynx 4.1 (Waters Corporation) software program. Raw data were processed using a Progenesis QI software (Non-linear Dynamics, Waters) with an in-built LipidBlast database (Kind et al., 2013) and LIPID MAPS Structure Database (RRID:SCR_003817) lipid identification. Identification of a lipid compound is based on the following main characteristics: accurate mass (ppm error <5), isotope pattern similarity (>80%), and fragmentation pattern. The lipid nomenclature and shorthand notation based on LIPID MAPS terminology and described by (Liebisch et al., 2013; Liebisch et al., 2020) were followed throughout this paper.

### 2.7 Targeted lipidomics

Targeted lipidomic analysis was performed on plasma and isolated liver microsomes of 24 male fish from Control and High-Dose groups (*n* = 8 per group) at Cinta Porte’s lab at CSIC, Spain, using Flow Injection Analysis High-Resolution Mass Spectrometry (FIA-HRMS). The fish used for targeted analysis included fish from the same groups used for untargeted analysis (see 2.6) but were otherwise randomly selected.

#### 2.7.1 Isolation of liver microsomes

Liver samples were homogenized as described by (Blanco et al., 2019), with some modifications. Homogenates were centrifuged at 1000 *g* for 5 min at 4°C. The supernatant was collected and further centrifuged at 12,000 x g for 45 min and 20,000 x g for 30 min. The obtained supernatant was centrifuged at 100,000 x g for 90 min. Microsomal pellets were then resuspended in a small volume of 100 mM phosphate buffer pH 7.4, 1 mM EDTA, 0.1 mM DTT, 0.1 mM PMSF and 20% w/v glycerol. Protein concentrations were determined by the method of Bradford, using bovine serum albumin as a standard (Bradford, 1976).

#### 2.7.2 Lipid extraction from plasma and liver microsomes

Lipids of 50 µL of plasma were extracted with ethyl acetate. Samples were incubated for 30 min at room temperature and soft shaking. The organic upper layer was collected, and the extraction was repeated twice. The final extract was evaporated under nitrogen. Microsomal lipids were extracted by a modification of (Folch et al., 1957). 200 μg protein were homogenized in ice-cold chloroform:methanol (2:1 v/v) containing 0.01% butylated hydroxytoluene (BHT). Samples were incubated for 30 min at room temperature and soft shaking; 0.88% KCl was added to the supernatant (1:4 v/v), thoroughly mixed and further incubated for 10 min. After centrifugation (2500 rpm, 10 min, 10°C) the organic layer was collected, the extraction was repeated, and the solvent evaporated under nitrogen.

#### 2.7.3 Instrumental analysis

The analysis of lipids was performed as described in (Vichi et al., 2012), with some modifications. Reconstituted lipid extracts were analyzed by direct injection in an Orbitrap-Exactive HCD mass spectrometer (Thermo Fisher Scientific, Bremen, Germany) equipped with heated electrospray source (H-ESI II), a Surveyor MS Plus pump and an Accela Open AS autosampler kept at 15°C (Thermo Fisher Scientific, San Jose, California). The mobile phase was methanol/dichloromethane 80:20 at 50 μL/min. Mass spectra were acquired in full scan both in ESI+ and ESI- ionization mode. The acquisition mass range was set to *m/z* 200–2000 and the total analysis time was 2 min. The ultrahigh resolving power defined as R = 100,000 (m/z 200, FWHM) was set. Mass peaks considered were single positive charged sodium molecular ions [M + Na^+^], including triacylglycerols (TG), diacylglycerols (DG), phosphatidylcholines (PC), their plasmanyl/plasmenyl forms (PCO/PCP), cholesteryl esters (CE); and single negative charge [M-H^+^], including phosphatidylethanolamines (PE), their plasmanyl/plasmenyl forms (PEO/PEP), phosphatidylserines (PS), phosphatidylinositols (PI), phosphatidylglycerols (PG), and free fatty acids (FA). Data from LIPID MAPS^®^, exact mass, isotopic distributions, charge, adducts formed, number of rings plus double bonds (RDB = 0.5–15) and elements in formula, were used for the identification of the lipid molecules with a maximum permitted mass error fixed at 5 ppm. Mass spectra were processed with Xcalibur (v2.1, Thermo Fisher Scientific, Bremen, Germany, RRID:SCR_014593) and lipid species were quantified with internal standards.

### 2.8 Statistical analyses

#### 2.8.1 Biometric analyses

Liver somatic index (LSI) was calculated as [liver weight (g, ww)/whole body weight (g, ww)] x100. Significant differences in LSI between controls (solvent exposed) and fish exposed for WY-14,643 and GW501516 were assessed with ANOVA and Dunnett´s multiple comparison test using GraphPad PRISM version 9 (RRID:SCR_002798).

#### 2.8.2 Ppar target genes involved in lipid metabolism

The statistical significance of the fold change of each gene and protein was calculated using independent two-sample t-tests between the control and response values. The *p*-values were adjusted within each gene set or pathway using the Bonferroni method (Bonferroni, 1936).

#### 2.8.3 Proteomics data analysis

In contrast to transcriptomics data, multiple testing correction was not performed on proteomics data. Due to the ratio compression effect (Bantscheff et al., 2012), TMT will report apparently smaller effects and higher *p*-values. Thus, calculation of false discovery rates (FDRs) in proteomics may result in few (if any) proteins pass the corrected thresholds, and should not be used bluntly (Pascovici et al., 2016). Hence, for the proteomics data, a list of differentially expressed proteins (DEPs) with less stringent cut off (*p* < 0.05 and fold-change cutoff 1.2) was used in the downstream analysis.

#### 2.8.4 Identifying statistically significant changes in liver lipid compounds following untargeted lipidomics

The fraction of each lipid compound among all measured lipids in every sample was calculated and was used in the downstream analysis. A linear model was fitted for each treatment group with the control group, and statistics were computed by empirical Bayes moderation of the standard errors using the R package “limma” (Ritchie et al., 2015). The Benjamini-Hochberg adjusted *p*-value of 0.05 was used as the threshold of significance for differential analysis.

#### 2.8.5 Univariate analysis and enrichment analysis of lipid profiles following targeted lipidomics

Univariate analysis of the lipid profiles was performed using the Metaboanalyst software version 3.0 (RRID:SCR_015539) (Xia et al., 2015). The missing values were first replaced using the KNN algorithm. Data were scaled by mean-centering and divided by the standard deviation of each variable (Xia and Wishart, 2016). Volcano plots based on fold-change values (>1.5) and a significance threshold of *p* < 0.05 (Student’s t-test) were used to visualize the significance and magnitude of the changes in the exposed groups compared to the control group. Enrichment analysis was performed using LION/web (RRID:SCR_017018) (Molenaar et al., 2019). Prior to this analysis, data were normalized, and the imputation of missing values was performed using half of the minimum value found in the dataset.

#### 2.8.6 Multi-omics data analysis

We performed a two-dimensional principal component analysis (PCA) for all complete datasets for biometrics, transcriptomics, proteomics, and lipidomics with no FDR cut-off, as well as for differentially regulated genes (DEGs, Supplementary Table S2), proteins (DEPs, Supplementary Table S3), and lipids (DALs, Supplementary Table S4). We show the fish projected to the two first principal components with 95% confidence ellipses for the different exposures and computed a Hotelling’s t-squared statistic to compare exposures (Hotelling, 1931).

## 3 Results

### 3.1 Atlantic cod peroxisome proliferator-activated receptors were transactivated *in vitro*

**FIGURE 1:**
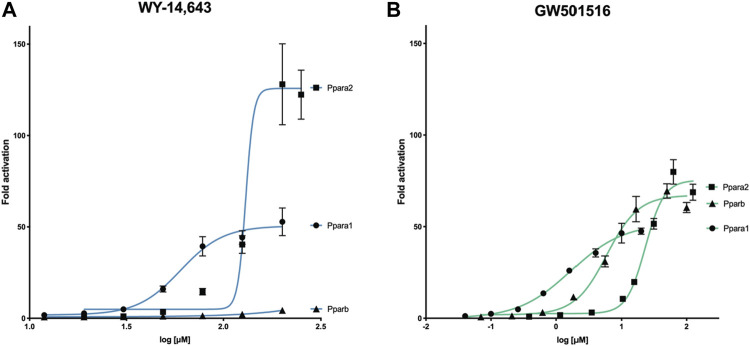
Activation of Atlantic cod Ppara1, Ppara2, and Pparb ligand-binding domain by **(A)** WY-14,643 and **(B)** GW501516 using luciferase reporter gene assays. COS-7 cells transfected with gmPpara1, gmPpara2, and gmPparb were exposed to either WY-14,643 or GW501516 at increasing concentrations. Concentration-response curves are presented as mean *±* SEM fold activation of the receptors in comparison to solvent control (DMSO). Data were recorded from three independent experiments with three technical replicates. Concentration-response curves were calculated by non-linear regression (4 parameters, GraphPad PRISM v. 9).

Using luciferase-based reporter gene assays with cod Ppara1, Ppara2, and Pparb, we demonstrated that WY-14,643 selectively activates Ppara1 and Ppara2 in a dose-dependent manner, but not Pparb (Figure 1A). The drug was more potent towards Ppara1 (EC_50_ = 60 μM) than Ppara2 (EC_50_ = 130 μM), whereas the efficacy was higher for Ppara2 (126 vs. 51 fold increase). In contrast, the mammalian PPARB agonist, GW501516, activated all three receptors (Figure 1B), but with less efficacy than WY-14,643 (max 75.3 μM for Ppara2). The EC_50_ ranged from 1.7 μM for Ppara1, 5.9 μM for Pparb, to 23.2 μM for Ppara2.

Our results showed that the GW501516 induced activation of the Ppars at about one-10th of the concentration of WY-14,643. Based on these findings, WY-14,643 were administrated at ten times higher exposure doses in the subsequent *in vivo* experiment.

### 3.2 *In vivo* treatment affected lipid metabolism at gene and protein levels

#### 3.2.1 No clear effects on biometric parameters

No significant effects were found on biometric parameters, such as the weight and length of male fish following the treatments in this study. Though the results indicated a slight increase in liver somatic index (LSI) in males following exposure to the low dose (6.5 ± 1.6) and high dose (6.8 ± 1.5) of GW501516, as well as high dose WY-14,643 (6.6 ± 1.0) in comparison to the control (6.0 ± 1.3), it was not statistically significant (Supplementary Figure S1).

#### 3.2.2 Low number of shared differentially expressed genes and proteins

**FIGURE 2:**
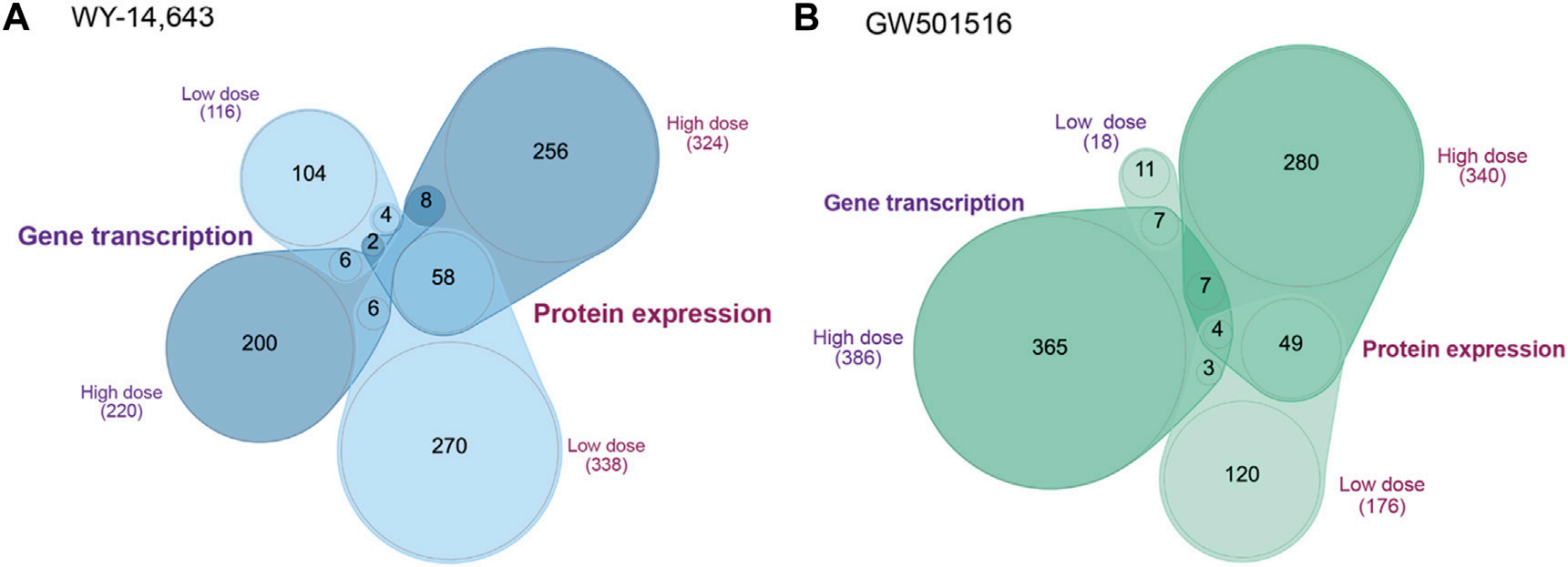
Quasi-proportional Venn diagram of shared differentially expressed genes and proteins in Atlantic cod liver following low and high dose of **(A)** WY-14,643 and **(B)** GW501516. Venn diagram was drawn based on the annotated ENSEMBL gene ID, using the nVenn tool (Perez-Silva et al., 2018).

Transcriptomics and proteomics datasets were obtained from male liver tissue samples. As shown in Figures 2A,B, the number of DEGs shared between low and high doses of WY-14,643 and GW501516 were low (6 and 7 genes, respectively), whereas the corresponding number of shared DEPs between low and high doses were higher (58 and 53 proteins, respectively). Comparing the number of shared DEGs and DEPs following exposure to low and high doses, we found 128 genes and 156 proteins affected by both compounds (Supplementary Figure S2). For the lists used in pathway analysis, additional cutoff values of at least 1.5 and 1.2 fold-changes were used for DEGs (Supplementary Table S2) and DEPs (Supplementary Table S3), respectively.

One-way hierarchical clustering analysis of the top DEGs from the different treatments grouped the genes into three main clusters (Supplementary Figure S3). Only a few genes shared similar expression profiles with slight upregulation with both chemicals, and these include genes coding for enzymes in lipid metabolism (SC5D, FABP1, and EBP) or drug metabolism (GSTP1 and NAT1). Notably, NAT1 (N-acetyltransferase 1) is an enzyme that is known to metabolize xenobiotics by transferring acetyl-CoA to arylamine and hydrazine substrates (Sim et al., 2008) also involved in the metabolism of WY-14,643 and GW501516.

#### 3.2.3 Significantly enriched pathways for WY-14,643 but not GW501516, based on transcriptomics data

Pathway and functional enrichment analyses were performed on the DEGs (FDR <0.05 and fold-change cutoff 1.5) derived from both low and high-dose treatments, using the ToppGene Suit (Chen et al., 2009). Exposure to WY-14,643 resulted in a list of significantly enriched pathways shown in Table 1. Pathways related to lipid metabolism were enriched, which included genes encoding enzymes in triglyceride biosynthesis (upregulated: *mogat2*, *acly*, *acot13* and *acsf2, and* downregulated: *fasn*, *lpin1*, and *lpin2*) (Table 1). Some of the DEGs in lipid metabolism pathways such as *mogat2*, *acot13*, *gos2,* and *lpin2* are known PPARA target genes in mammals (Rakhshandehroo et al., 2010). Moreover, LPIN1 and LPIN2 are in mammalian species involved in glycerolipid biosynthesis (Reue and Zhang, 2008), and the downregulation of their genes, as well as the gene coding for FASN, appears to be consistent with reduced triglyceride levels. Furthermore, the enzymes ACOT13 and ACSF2 are involved in lipid degradation in mammals (Hunt et al., 2012; Watkins and Ellis, 2012) and the upregulation of these genes suggests decreasing effects on plasma triglyceride levels by WY-14,643 in cod.

**TABLE 1:** Significantly enriched (FDR q value < 0.05) pathways in differentially expressed genes from treatment with WY-14,643. Gene lists from low and high-dose treatments were combined in the analysis. Analysis was performed using ToppGene Suite.

Pathway name	Source	Hits in Query list
**Triglyceride Biosynthesis**	REACTOME	MOGAT2, ACLY, ACOT13, ACSF2, LPIN1, FASN, LPIN2
**Adipogenesis**	MSigDB C2 BIOCARTA	IRS4, NR3C1, GADD45A, DDIT3, LPIN1, IGF1, LMNA, LPIN2
**Terminal pathway of complement**	REACTOME	CLU, C7, C9
**Cholesterol metabolism (includes both Bloch and Kandutsch-Russell pathways)**	MSigDB C2 BIOCARTA	CYP46A1, FASN, EBP, SOAT2, SOAT1
**Insulin/IGF pathway-MAP kinase cascade**	PantherDB	IRS4, IGF1, ELK1, MAP2K1
**Pathways in clear cell renal cell carcinoma**	MSigDB C2 BIOCARTA	DEPTOR, RAPGEF5, ACLY, CAMK1, FASN, ALDOC
**ATF-2 transcription factor network**	Pathway Interaction Database	DUSP8, GADD45A, DDIT3, HES1, PLAU
**Nuclear Receptors Meta-Pathway**	MSigDB C2 BIOCART	SLC39A2, ENC1, NR3C1, PTGS2, FABP1, FASN, SERPINB9, IGFBP1, HES1, PLTP, ALOX5AP
**ATF-2 transcription factor network**	MSigDB C2 BIOCARTA	DUSP8, GADD45A, DDIT3, HES1, PLAU
**Ghrelin: Regulation of Food Intake and Energy Homeostasis**	MSigDB C2 BIOCARTA	RAPGEF5, IGF1, IGFBP1
**Prion diseases**	MSigDB C2 BIOCARTA	ELK1, C7, C9, MAP2K1
**Prion diseases**	KEGG	ELK1, C7, C9, MAP2K1
**Depolymerisation of the Nuclear Lamina**	REACTOME	LPIN1, LMNA, LPIN2
**Genes related to PIP3 signaling in cardiac myocytes**	MSigDB C2 BIOCARTA	IRS4, GADD45A, IGF1, IGFBP1, EBP
**Metabolism of lipids and lipoproteins**	REACTOME	MOGAT2, ACLY, ACOT13, TPTE2, G0S2, ACSF2, FABP12, CYP46A1, PTGS2, FABP1, LPIN1, FASN, EBP, LPIN2, PLTP, ALOX5AP, SOAT2, SOAT1
**Glucocorticoid Receptor Pathway**	MSigDB C2 BIOCARTA	ENC1, NR3C1, PTGS2, SERPINB9, ALOX5AP
**Steroid biosynthesis**	MSigDB C2 BIOCARTA	EBP, SOAT2, SOAT1
**Fatty acid, triacylglycerol, and ketone body metabolism**	REACTOME	MOGAT2, ACLY, ACOT13, G0S2, ACSF2, FABP1, LPIN1, FASN, LPIN2
**Androgen/estrogene/progesterone biosynthesis**	PantherDB	SOAT2, SOAT1
**Serotonin Receptor 4/6/7 and NR3C Signaling**	MSigDB C2 BIOCARTA	NR3C1, ELK1, MAP2K1
**Steroid biosynthesis**	KEGG	EBP, SOAT2, SOAT1
**Oxidative stress response**	PantherDB	MKNK2, DUSP8, DDIT3, ELK1
**IGF-1 Signaling Pathway**	MSigDB C2 BIOCARTA	IGF1, ELK1, MAP2K1

On the other hand, the lipogenic genes *mogat2* and *acly* were also upregulated. Although PPARA ligands primarily promote lipid degradation in mammals, it is also known that some lipid biosynthesis pathway genes are PPARA targets (Rakhshandehroo et al., 2010). The effects on lipid metabolism pathways were further reflected in the significantly enriched (ToppGene Suit, FDR q-value <0.05) diseases terms (DisGeNET database) phenotypes such as “Fatty Liver” and “Steatohepatitis” (Supplementary Table S5).

In contrast to the effects seen from exposure to WY-14,643, similar pathway enrichment analysis did not result in any significantly enriched pathways for GW501516 (not shown).

#### 3.2.4 Pathway analysis of differentially expressed proteins indicate that WY-14,643 affect lipid metabolism also at the protein level

**FIGURE 3:**
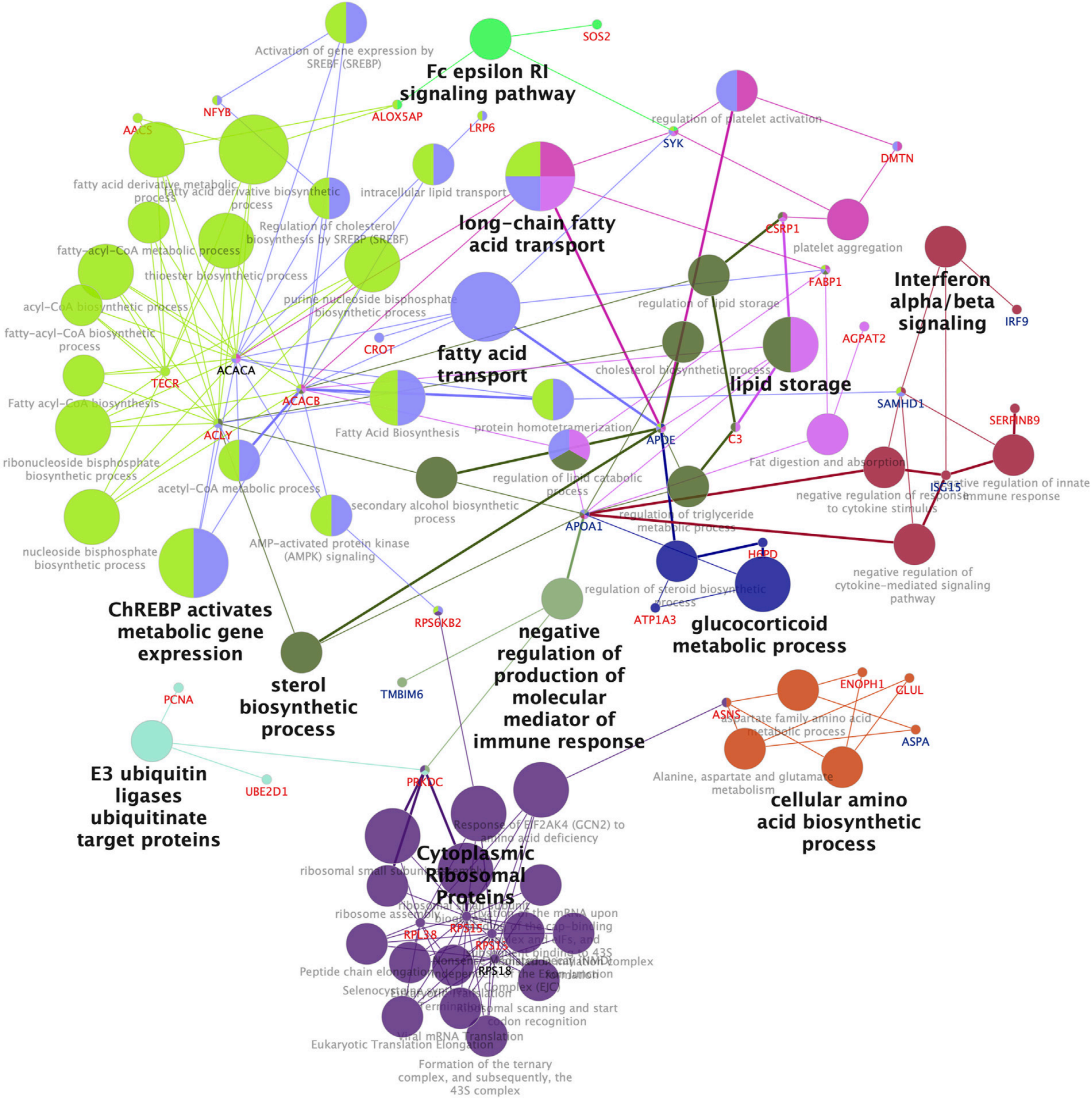
Enriched pathways and networks in proteins differentially expressed in the liver of Atlantic cod injected with WY-14,643. Enrichment of KEGG pathways, Wikipathway, Reactome and Gene Ontology Biological Processes was performed in Cytoscape using the ClueGo application. Functionally related pathway terms (nodes) are grouped by same color, and the label of the most significant term is highlighted. The node size is proportional to the number of constituent proteins. Only significantly enriched networks (Bonferroni step-down correction, adjusted *p*-value <0.05) are shown. Protein symbols are colored red and blue for up- and downregulated proteins, respectively.

Pathway analysis performed in Cytoscape using the ClueGo application (Shannon et al., 2003; Bindea et al., 2009) for DEPs following treatment to WY-14,643 (combined list of high dose and low dose), resulted in significantly enriched (FDR <0.05) networks and pathways (Figure 3). Among the DEPs are enzymes involved in the regulation of fatty acid synthesis and beta-oxidation in mammals, such as ACACA, ACACB, and CROT (Schreurs et al., 2010) (Figure 3). Many of the proteins in the lipid metabolism pathway were upregulated, except Apoe and Apoa1, which were downregulated (Figure 3). The genes encoding some of the lipid metabolism pathway enzymes such as ACACA and CROT (Figure 3) are known PPARA targets in mammals (Rakhshandehroo et al., 2010), which is consistent with the upregulation of their protein products in WY-14,643 treated cod liver. Similarly, significantly enriched GO biological processes following treatment by WY-14,643 show many DEPs involved in lipid metabolism (Supplementary Table S6).

For DEPs following injection to GW501516 (combined list of low dose and high dose), the top enriched biological processes are mainly related to protein translation. However, GO BP “triglyceride catabolic process”, populated by three proteins (APOA1, FABP1, and FABP7), was significantly enriched (Supplementary Table S7).

#### 3.2.5 Transcription of Ppar target genes involved in lipid metabolism and transport were affected by the treatments

Strong upregulation of genes involved in fatty acid beta-oxidation (Supplementary Figure S4A), known as one of the primary target processes under PPARA regulation in mammalian species, was not observed by the Ppara ligand WY-14,643 in this experiment. The high dose of GW501516 significantly downregulated transcription of acetyl-CoA acyltransferase1 (*acaa1*) and enoyl-CoA hydratase/3-hydroxyacyl-CoA dehydrogenase (*ehhadh*), indicating that the last steps of the β-oxidation were affected by this drug in cod.

As for genes involved in fatty acid transport (Supplementary Figure S4B), the results showed that high dose WY-14,643 upregulated fatty acid-binding proteins, such as the liver-specific *fabp1b.1*, the muscle and heart-specific *fabp3*, the brain-specific *fabp7*, and the fish-specific *fabp11a*. Here, GW501516 only upregulated transcription of the liver-specific *fabp10a* and *fabp11a*.

When looking at genes involved in the extracellular transport of lipids, including transporters of TG, phospholipids, and cholesterol esters (Supplementary Figure S4C), and apolipoproteins (Supplementary Figure S4D), there was high variation between the fish individuals, also in the control group. However, all treatments significantly downregulated low-density lipoprotein receptor (*lplra*). In addition, the high dose of WY-14,643 downregulated the cholesterol efflux regulator (*abca1*) and lipoprotein lipase (*lpl*), and the low dose significantly downregulated the phospholipid transporter protein (*pltp*), which together indicate a reduced lipoprotein uptake. Treatment with high dose GW501516, on the other hand, showed increased transcription of apolipoproteins (*apoa4b.2* and *apol1*), indicating an increase in extracellular transport of lipids.

The protein levels of these Ppar target genes were not significantly affected, except for *fabp1b.1* and *fabp11a* that were slightly upregulated following exposure to high dose GW501516 (not shown). Interestingly, transcriptional and translational levels of ATP-citrate lyase (*acly*) significantly decreased following treatment with high dose WY-14,643. Although not considered a target gene for PPARA, but rather PPARB, in rodents, this enzyme is responsible for metabolizing citrate to acetyl-CoA in the initial step of the fatty acid synthesis, which could indicate a shift in energy production through the citric acid cycle.

### 3.3 *In vivo* activation of Atlantic cod Ppars affect lipid composition in plasma and hepatic microsomes

#### 3.3.1 Whole liver lipids: Untargeted lipidomics analysis showed effects on lipid compounds of different classes

**FIGURE 4:**
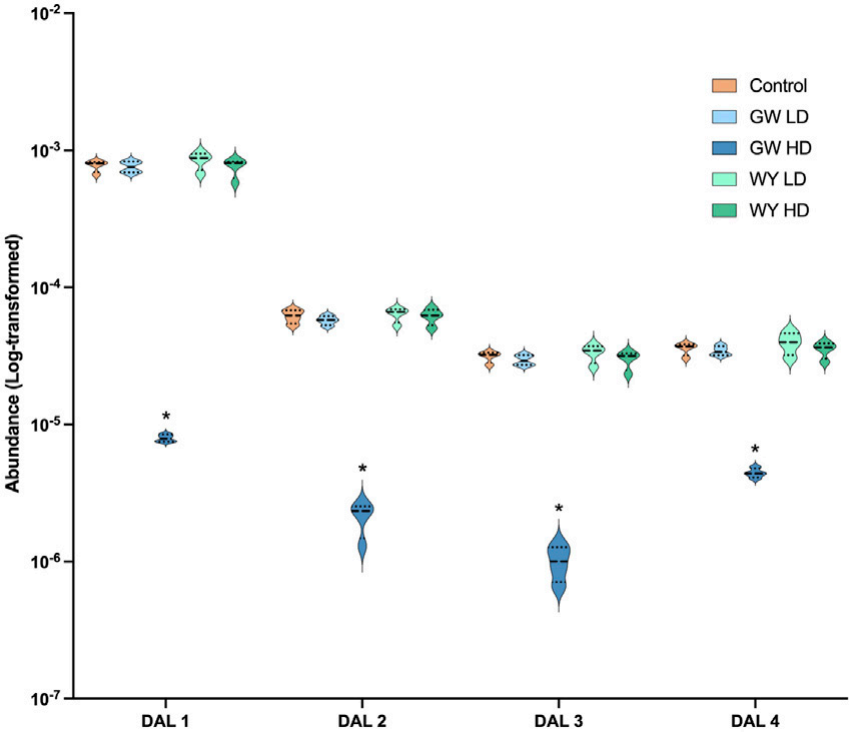
Changes in significantly affected lipids in Atlantic cod whole liver tissue following exposure to WY-14,643 and GW501516. The levels of differentially abundant lipids (DALs) were measured using untargeted UPLC-HDMS lipidomics (see Supplementary Table S4 for DAL ID characterization). Lipid compounds significantly (adjusted *p* < 0.05) affected by the exposures were identified using “limma” and are indicated by an asterisk (*). Violin plots with median (solid line) and quartiles (dotted lines) indicated were produced in GraphPad PRISM v.9.

Levels of lipid compounds in whole liver tissue were analyzed using UPLC-HDMS. A total of four compounds were found differentially regulated following exposure to high dose GW501516. All of these (putatively three diglycerides and one monoglyceride) were downregulated (Supplementary Table S4). Neither low dose GW501516 nor low or high dose WY-14,643 had any significant effect on these compounds (data summarized in Figure 4).

#### 3.3.2 Microsomal lipids: Targeted lipidomics analysis revealed increase in phospholipids and downregulation of TGs

**FIGURE 5:**
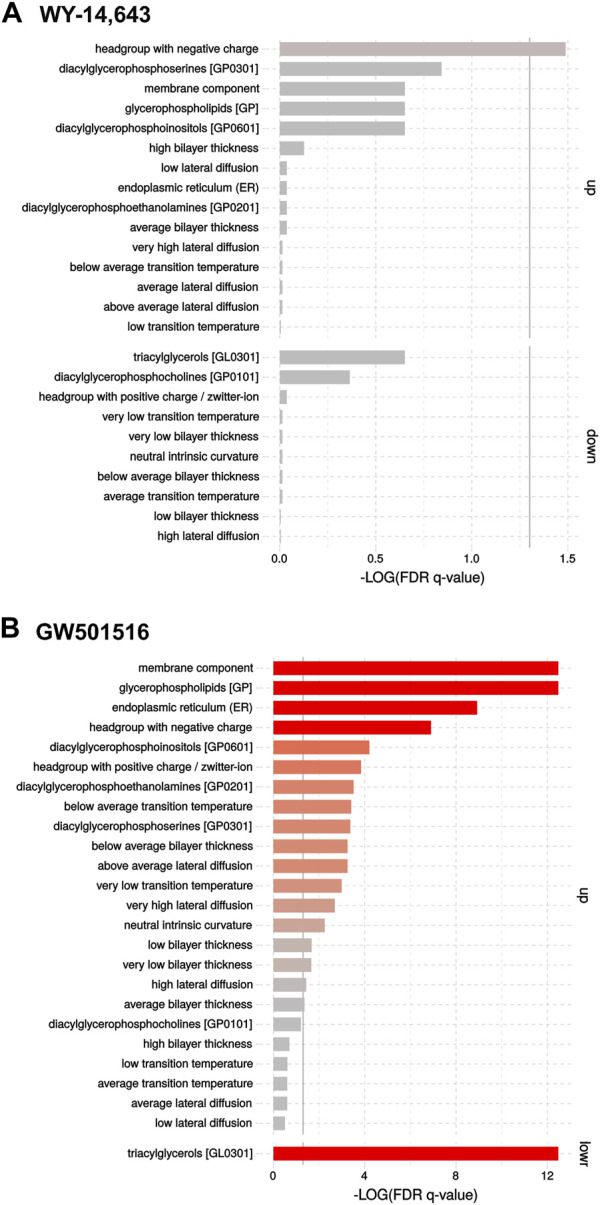
Enrichment analyses of FIA-HRMS lipidomic data in cod liver microsomes following treatment with the high dose of **(A)** WY-14,643 and **(B)** GW501516. The analyses were performed on normalized targeted lipidomics data, using the LION/web tool (Molenaar et al., 2019).

Changes caused by the treatments on lipidomic profiles of the isolated microsomal fraction of liver cells using FIA-HRMS are shown in the Volcano plots (Supplementary Figure S5). In short, exposure to high dose WY-14,643 lead to a significant increase in the phosphatidylinositol PI40:7 and the phosphatidylethanolamines PE42:6 and PE38:4. Effects from exposure to high dose GW501516 showed a generalized decrease of TGs and a concomitant increase of PI, PE and PS containing PUFAs (PE38:4, PE38:7, PE40:8, PE42:6, PS38:5, PS40:6, PI36:5, PI38:5). Enrichment analysis (Figure 5) showed the changes more clearly: Exposure to WY-14,643 slightly increased levels of lipids with “headgroup with negative charge” (Figure 5A), whereas GW501516 strongly increased “membrane components” and “glycerophospholipids” such as “diacylglycerophosphoinositols” (PI), “diacylglycerophosphoethanolamines” (PE) and ‘diacylglycerophosphoserines’ (PS), and strongly downregulated “triacylglycerols” (Figure 5B).

#### 3.3.3 Plasma lipids: Targeted lipidomics analysis revealed decrease in phospholipids and mixed regulation of TGs

**FIGURE 6:**
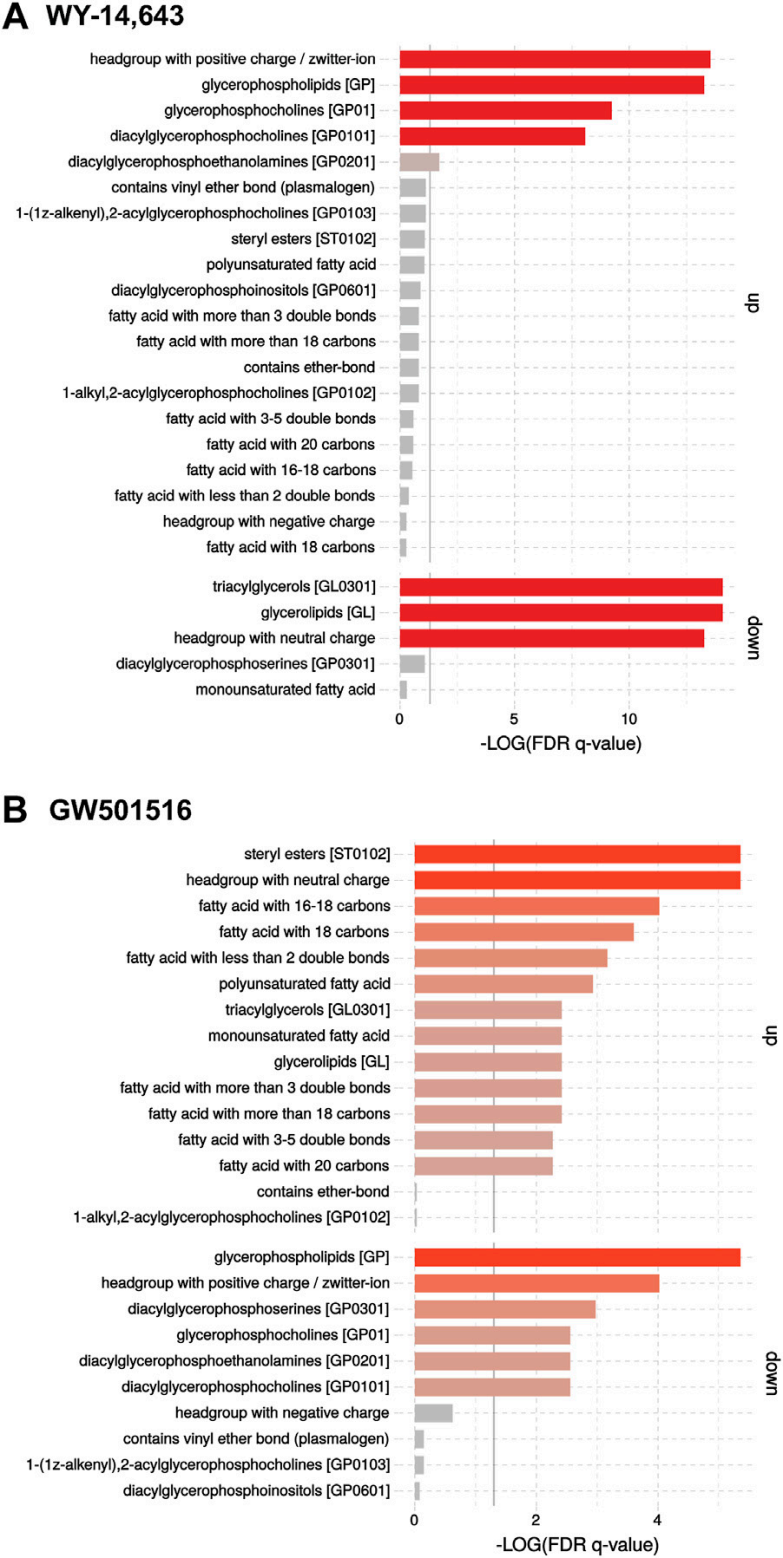
Enrichment analyses of FIA-HRMS lipidomic data in cod plasma following treatment with **(A)** WY-14,643 and **(B)** GW501516. The analyses were performed on normalized targeted lipidomics data, using the LION/web tool (Molenaar et al., 2019).

Lipidomics analysis of the plasma using FIA-HRMS found effects contrasting those seen in the microsomal fraction, as shown in the Volcano plots (Supplementary Figure S6). Results show that high dose WY-14,643 resulted in a significant decrease in PI40:3 and the phosphatidylserine PS38:4, as well as a trend towards a decrease in TGs in plasma. GW501516 only caused significantly decreased levels of PI40:3 and phosphatidylcholine PC38:8. Enrichment analysis indicated again a stronger response than found in the univariate analysis: In plasma, WY-14,643 increased levels of lipids with “headgroups with positive charge” and “glycerophospholipids” (PC, PE, PI), and strongly decreased TGs (Figure 6A), thus seemingly having a stronger effect in plasma than in the microsomal fraction. GW501516, on the other hand, had less effect in plasma compared to microsomes, but enrichment analyses revealed an increased abundance of CE and TG in plasma, which was not highlighted by the univariate analysis (Figure 6B). Furthermore, an enrichment of lipids containing fatty acids with 16–18 C and fatty acids with less than two double bonds, as well as polyunsaturated fatty acids, were found.

### 3.4 Multi-omics data analysis

**FIGURE 7:**
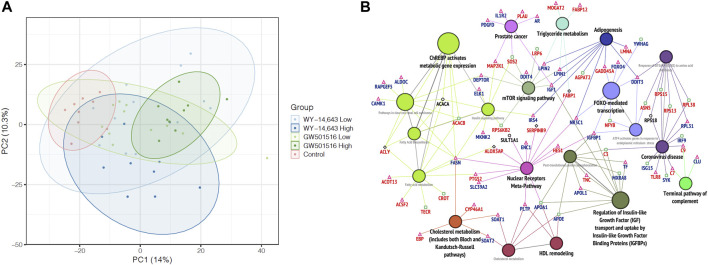
Multi-omics analyses. **(A)** Principal component analysis of differentially affected genes, proteins, and lipids. The graph shows data points and 95% confidence ellipses for each treatment group. **(B)** Integrative analysis of pathways and networks using merged lists of differentially expressed genes and proteins in Atlantic cod liver after WY-14,643 treatment, using ClueGo in Cytoscape. Significantly enriched networks (adjusted *p*-value < 0.05) generated using ClueGo with Wikipathway, KEGG and Reactome databases are shown. Gene/protein symbols shown in red and blue indicate upregulation and downregulation, respectively. Triangle, square and diamond symbols, represent transcripts, proteins and both (transcripts and proteins), respectively. For a similar analysis of GW501516 treatment, see Supplementary Figure S7.

Using principal component analysis (PCA) of the complete datasets with no FDR threshold, no separation of the different exposure groups was observed at any level of investigation (results not shown). As not all samples (fish) were analyzed in all omics methods, an integration of all available results into one analysis was not feasible. Taking a more targeted approach, we performed a new PCA using only the differentially affected genes (DEGs), proteins (DEPs), and lipids (DALs). This time, a clear separation of the high dose GW501516 fish from the control group was observed, displaying an apparent dose-response trend (Figure 7A). On the other hand, high dose WY-14,643 tended to pull the fish in a different direction, although this was not statistically significant.

To explore these differences further, we returned to an integrated pathway analysis of the transcriptome and proteome data using Cytoscape. In the WY-14,643 treated fish, visualization of the resulting enriched pathways in ClueGo, revealed a more elaborate effect on the lipid homeostasis. Similar to the findings in single-omics analysis of WY-14,643 treated fish, most enriched pathways were related to lipid metabolism, but new genes and proteins were also highlighted using the integrated approach. Particularly, WY-14,643 increased the levels of many lipid catabolic transcripts (e.g., *actot13, acsf2*) and enzymes (e.g., Tecr, Crot), suggesting increased lipid degradation (Figure 7B), which appears to be consistent with the decreased plasma TG levels (Figure 6A). In parallel, lipid synthesis and efflux related genes such as *lpin1*, *lpin2*, *soat1*, *soat2*, and *fasn* were downregulated either at the transcript or protein levels (Figure 7B), suggesting a decreased lipid synthesis. However, some genes or protein products that seem to be lipogenic were upregulated, including *acly* (increased at both transcript and protein levels), *mogat2* (increased at transcript levels), and Acacb (increased at protein levels) (Figure 7B). In the GW501516 treated fish, on the other hand, only a few lipid metabolism related genes and pathways were enriched (Supplementary Figure S7) and gave little new information compared to the single-omics analyses.

## 4 Discussion

Understanding the regulation of lipid metabolism of marine fish species is important, not only for understanding fish physiology and energy homeostasis, but also from a human nutrition and environmental risk assessment perspective. The Atlantic cod liver stores 50%–70% of the total lipid content as lipid droplets within the cytoplasm of hepatocytes (Jangaard et al., 1967), giving cod hepatocytes a combined function of hepatic metabolism and fat storage. Although studies have described the lipid levels at different developmental stages and following different feeding regimes in Atlantic cod (Lie and Lambertsen, 1985; Lie et al., 1986; Lie et al., 1988), the regulatory mechanisms of lipid metabolism and the role of Ppars are not well studied. Ppars are of particular interest due to the increasing number of chemicals released into the environment that may act through these receptors, such as fibrate drugs, phthalates, and perfluorinated compounds (Heindel and Blumberg, 2019), and potentially alter the lipid regulation in cod, and thus, its nutritional benefits for humans. In this study, we have investigated the regulation of lipid metabolism following activation of Ppara and Pparb in juvenile Atlantic cod.

### 4.1 WY-14,643 specifically activated Ppara1 and Ppara2 *in vitro* and induced a shift in lipid transport *in vivo*

The role of PPARA in peroxisomal fatty acid β-oxidation is well established in some mammalian species (Desvergne and Wahli, 1999; Mandard et al., 2004). WY-14,643 is a specific activator of mammalian PPARA and induces hepatic PPARA target genes and metabolic responses (Kliewer et al., 1994; Li et al., 2018; Strand et al., 2019). Although there are a few studies describing responses of WY-14,643 in marine fish *in vivo*, namely, turbot (*Scophthalmus maximus,* Scophthalmidae) and polar cod (*Boreogadus saida,* Gadidae) (Urbatzka et al., 2015; Vieweg et al., 2018), the mechanisms and metabolic outcomes in fish are less understood.

As demonstrated previously (Søderstrøm et al., 2022), WY-14,643 specifically activated cod Ppara1 and Ppara2, but not Pparb *in vitro*. The compound was most potent towards Ppara1, but Ppara2 reached the highest activation. Similarly, WY-14,643 specifically activated Ppara in sea bream and plaice *in vitro* (Leaver et al., 2005). Thus, the specificity of WY-14,643 to exclusively activate Ppara seems to be retained in fish. However, our study indicates that, compared to rodents, WY-14,643 is a weaker activator of the fatty acid β-oxidation pathway, but rather induces a shift in lipid transport in Atlantic cod.

Omics analysis of Atlantic cod exposed to WY-14,643 *in vivo* showed effects on lipid metabolism pathways, including many genes known to be PPARA targets in mammals (Rakhshandehroo et al., 2010). In contrast to what is known from mammalian studies, we did not find a significant induction of genes in lipid degradation pathways such as fatty acid β-oxidation. For example, the cod ortholog of the established biomarker target gene for PPARA-activation in mammals, *ACOX* (Mandard et al., 2004), remained unaffected by exposure to WY-14,643 in our *in vivo* study. Ppara-induced responses in fish, including induction of *acox*, is previously found to be time- and tissue-specific (Mortensen et al., 2011; Urbatzka et al., 2015), or even unaffected by Ppara agonists (Madureira et al., 2017; Vieweg et al., 2018) which may be partially driven by the high clearance rate of fibrates in teleosts (Mimeault et al., 2005). However, ACOX gene expression was not significantly affected in response to fibrate treatment in human liver (Roglans et al., 2002), suggesting species differences in some of the responses.

We have recently shown that Ppara pathways such as β-oxidation can be activated by perfluoroalkyl substances (PFAS) in Atlantic cod following 7 days of exposure (Dale et al., 2020). Here, we also sampled 7 days after the second dosing, but compared to PFAS which have long half-lives *in vivo* (Goeritz et al., 2013), the compounds used here may have been cleared from the cod tissue more rapidly. Thus, we might have missed the transient induction of genes involved in β-oxidation, and rather observed secondary transcriptional responses and increased synthesis of enzymes involved in lipid degradation such as Crot and Acab. In another study, we observed activation of Ppara pathway responses during vitellogenesis-related lipid mobilization in the liver of xenoestrogen treated Atlantic cod (Yadetie et al., ms in preparation), presumably related to changes in the levels of lipids able to modulate the activity of these receptors (Hihi et al., 2002).

While lipid degradation pathways appeared not to be activated at our sampling time point, our results showed that the transport of fatty acids was affected. The low-density lipoprotein receptor (*ldlrb*), which plays a critical role in endocytosis of lipids from plasma (Jeon and Blacklow, 2005), and the lipoprotein lipase (*lpl*), which hydrolyzes triglycerides (TG) to free fatty acids and VLDL to LDL (Eckel and Wang, 2009), were downregulated by WY-14,643. Furthermore, several fatty acid-binding proteins (Fabps), including *fabp1b.1*, *fabp3*, *fabp7*, and *fabp11a*, were induced. As FABPs are considered responsible for the intracellular transport of fatty acids to the endoplasmic reticulum (Schroeder et al., 2010), this could indicate a shift from the uptake of circulating plasma lipids to the use of intracellular fatty acids (Urbatzka et al., 2015).

The shift in lipid transport was further supported by lipidomics analysis, where a decrease in TG levels in plasma following exposure to high dose WY-14,643 was observed. Together with hydrophobic cholesteryl esters (CE) and phospholipids (PL), TGs make up the main components of plasma lipoproteins in both mammals and Atlantic cod (Li et al., 2016; Pan and Segrest, 2016). A decrease in plasma TG levels thus indicates a decrease in lipoprotein circulation. Notably, strong induction of lipid catabolism pathway genes and proteins were not observed, which might be attributed to our study design where peak changes in transcriptomics and proteomics responses may have diminished after 7 days of treatment. However, changes in lipid profile are expected to come later than the gene or protein expression changes, and lipidomics results thus appear to better reflect the systemic effects of the compounds. The changes in plasma and liver TG levels are mostly consistent with the pharmacological effects of WY-14,643 and GW501516 that are known plasma triglyceride-lowering drugs from mammalian studies (Evans et al., 2004). One surprising exception is our finding of increased TG levels in plasma after GW501516 treatment.

At the same time, the increase in some microsomal membrane phospholipids (phosphatidylethanolamines; PEs and phosphatidylinositols; PIs) can perhaps be explained by the increased levels of Fabps. Phospholipids include negatively charged molecules (i.e., PI and phosphatidylserines; PS), or lipids with zwitter-ion headgroups such as phosphatidylcholines (PC) and PE. The membrane surface charge depends on the membrane’s content of negatively charged phospholipids, which mediates functional interactions with positively charged regions of peripheral and integral membrane proteins (de Kroon et al., 2013). An induced shift in membrane surface charge could thus have subsequent adverse signaling effects.

### 4.2 GW501516 activate cod Ppara1, Ppara2 and pparb in a dose-dependent manner *in vitro* and induce lipoprotein transport and decreases in TG levels *in vivo*

In mammals, high-affinity PPARB ligands have revealed an important role in hepatic glucose utilization and lipoprotein metabolism, as well as an anti-inflammatory role (Oliver et al., 2001; Dressel et al., 2003). GW501516 was developed as a PPARB-selective agonist and is specifically shown to cause lipid catabolism and cholesterol transport out from mammalian cells (Brunmair et al., 2006). To our knowledge, this ligand has previously only been used in one study with teleost fish. There, one of the four Pparb isotypes in Atlantic salmon (*S. salar*) was found to be activated by GW501516, but not by WY-14,643 (Leaver et al., 2007).

The results from our *in vitro* reporter assays, however, showed that GW501516 activates both Pparb, Ppara1, and Ppara2 in Atlantic cod. Compared to WY-14,643, GW501516 is in fact more potent to Ppara1 and produces higher efficacy to Ppara2. Although the nuclear receptor superfamily is highly conserved in vertebrate evolution, inter-species variation in receptor activation and responses is well documented (Carvan et al., 2007; Zhao et al., 2015). We have recently cloned and characterized the Atlantic cod Ppars, demonstrating amino acid differences in the ligand-binding domains that could explain differences in ligand specificity between cod and human orthologs (Søderstrøm et al., 2022). Furthermore, we showed that the level of *ppara1* mRNA is about five times higher than *pparb* and ten times higher than *ppara2* in Atlantic cod liver tissue (ibid.) Whether the downstream effects from exposure to GW501516 in Atlantic cod *in vivo* are mainly caused by Ppara or Pparb, or both, require further investigations involving gene knockouts or knockdowns, experiments that are difficult to perform in cold water marine teleost species with a long reproductive cycle such as Atlantic cod.

The *in vivo* exposure to GW501516 did not result in significant enrichment of pathways related to lipid metabolism at the transcriptomic level in Atlantic cod. Still, GW501516 induced transcription of multiple Ppar target genes, including several fabp (*fabp10a* and *fabp11a*) and apolipoproteins (*apoa4b.2* and *apol1*), and caused downregulation of the low-density lipoprotein receptor (*lplra*), indicating an increased transport of fatty acids and lipids. However, multiple testing on proteomics data did not reveal any statistically significant changes in protein levels following exposure to either WY-14,643 or GW501516.

In contrast to transcriptomics data analysis, multiple testing on proteomics data is suggested to be less effective, especially in low power situations (Pascovici et al., 2016). Thus, we used the lists of DEPs with *p* < 0.05 in subsequent pathway analysis, where three DEPs (APOA1, FABP1and FABP7) resulted in significant enrichment of the GO triglyceride catabolic process, suggesting effects of GW501516 on lipid mobilization in cod. In early larvae stages of turbot (*S. maximus*), transcript levels of apolipoproteins and other lipid transporters are suggested to be regulated by Ppara2, whereas extracellular lipid transfer proteins are regulated by Ppara1 (Cunha et al., 2015). None of the genes were found correlating with Pparb mRNA levels (Cunha et al., 2015). Similarly, in a study using PPARA −/− and PPARB/D −/− mice, they found that the receptors are equally important in the fed state, with mildly overlapping liver gene regulation, but in the fasting state, the deletion of PPARA proved more dramatic than that of PPARB/D (Sanderson et al., 2010).

While the specific Ppar and signaling pathways through which GW501516 initiates its effects in Atlantic cod need further investigation, the exposure caused changes in lipid composition in plasma and liver microsomes. The upregulation of highly unsaturated membrane lipids of the microsomal fraction can enable density adjustments across the bilayer by creating a third dimension of fluidity in a structure that is essentially fluid in the x-y plane (Barelli and Antonny, 2016).

The decreases in stored liver TGs in the WY-14,643 and GW501516 exposed cod is consistent with the lipid catabolism promoting effects of these compounds in mammals (Evans et al., 2004). Also, the observed decrease in TGs in liver microsomes might indicate the use of these as a source of precursors for the synthesis of the highly unsaturated membrane phospholipids. As a result, the alterations in lipids and lipid-related cellular components in the microsomal fraction following treatment with GW501516, and to a lesser extent WY-14,643, could result in a dysregulation of the physicochemical properties and functions of ER membranes.

### 4.3 Multi-omics analysis of WY-14,643 effects suggests conserved PPARA functions

Although the WY-14,643 responses in cod appeared to be weak as discussed above, the integrated transcriptomics and proteomics data showed enhanced effects of the treatment on lipid metabolism related pathways, largely consistent with the pharmacological effects of PPARA agonists in mammals. Particularly, the PPARA target lipid catabolism related pathways, combined with lowered plasma TG levels seen in the lipidomics analyses, were consistent with mammalian effects. WY-14,643 and other PPARA agonists such as fibrates are known to modulate many genes and processes related to lipid metabolism including fatty-acid beta oxidation, synthesis and breakdown of triglycerides, lipoprotein metabolism and various other metabolic pathways (Kersten and Stienstra, 2017; Bougarne et al., 2018). The effects of PPARA agonists are less studied in fish, and our multi-omics analysis here strongly suggests that the functions of PPARA in mediating lipid metabolic processes are conserved in fish.

## 5 Conclusion

By using *in vitro* and *in vivo* approaches, we have shown that WY-14,643 and GW501516 activate Atlantic cod Ppars, affect genes in lipid metabolism pathways, and induce changes in the lipid composition of plasma and liver, providing insight in the physiological regulation of lipid metabolism through these receptors. The alterations in the lipid system observed with WY-14,643 and GW501516 indicate that other chemicals with similar Ppar receptor affinities might cause disturbances of lipid regulations in fish, as recently suggested for PFAS by Yang et al. (2023). However, our laboratory exposure study here focused on mechanistic aspects of PPAR agonist effects on lipid metabolism in cod, and our results are thus only suggestive of potential environmental risks of such compounds that should be further investigated.

In general, our omics analyses appear to reflect the species-specific differences in responses to PPARA ligands previously shown in vertebrates, with rodents eliciting stronger responses compared to primates and fish (Klaunig et al., 2003; Urbatzka et al., 2015). Still, clear distinctions were seen in lipid types between liver microsomes and plasma lipids, with both compounds causing increased levels of some phospholipids in liver and decreased levels in plasma. TGs decreased in stored liver lipids and in liver microsomes, more clearly after GW501516 treatment, whereas in plasma the TG response was opposite between the two compounds, with a decrease with WY-14,643 and a surprising increase with GW501516. Despite the species differences, our results, taken together with these earlier studies, indicate that the PPARA and its ligands share similarities in their effects on lipid metabolism in fish and other vertebrates (Rakhshandehroo et al., 2010; Urbatzka et al., 2015; Dale et al., 2020). In contrast, the ligand GW501516, specific for PPARB in rodents, activated both Atlantic cod Ppara1, Ppara2, and Pparb. The effects from this ligand on activation of cod Pparb were thus less explicit.

Though the underlying mechanisms remain elusive, the experiences from our integrative approach and the resulting multi-omics dataset provide novel insights into the systemic regulation of lipid metabolism in Atlantic cod, a species with a highly specialized lipid storage system. Ultimately, such data will broaden our understanding of lipid homeostasis in teleosts and their vulnerability to pollutants targeting the Ppar receptors.

## Data Availability

The original contributions presented in the study are publicly available. This data can be found here: https://doi.org/10.15490/fairdomhub.1.study.802.3. Biometric data and untargeted lipidomics data can be found here: https://doi.org/10.15490/fairdomhub.1.datafile.3950.1 and https://doi.org/10.15490/fairdomhub.1.assay.1646.1. The other omics datasets presented in this study can be found in online repositories. The names of the repositories and accession numbers can be found below: https://www.ebi.ac.uk/arrayexpress/, E-MTAB-11490, http://www.proteomexchange.org/, PXD031739, https://www.ebi.ac.uk/metabolights/, MTBLS2131.
