# Supplementary material for: Integrative omics-analysis of lipid metabolism regulation by peroxisome proliferator-activated receptor a and b agonists in male Atlantic cod
Source: Front Physiol. 2023 Mar 22;14:1129089. doi: 10.3389/fphys.2023.1129089 (PMC10073473; doi:10.3389/fphys.2023.1129089)
Supplement: Supplementary file 1 [file DataSheet1.docx]

## *Supplementary material*

## Integrative omics-analysis of lipid metabolism regulation

## by peroxisome proliferator-activated receptor a and b agonists

## in male Atlantic cod (*Gadus morhua*)

Marta Eide, Anders Goksøyr*, Fekadu Yadetie, Alejandra Gilabert, Zdenka Bartosova, Håvard G. Frøysa, Shirin Fallahi, Xiaokang Zhang, Nello Blaser, Inge Jonassen, Per Bruheim, Guttorm Alendal, Morten Brun, Cinta Porte, Odd André Karlsen

* Correspondence: Corresponding author: e-mail: anders.goksoyr@uib.no

**1 Supplementary Figures**


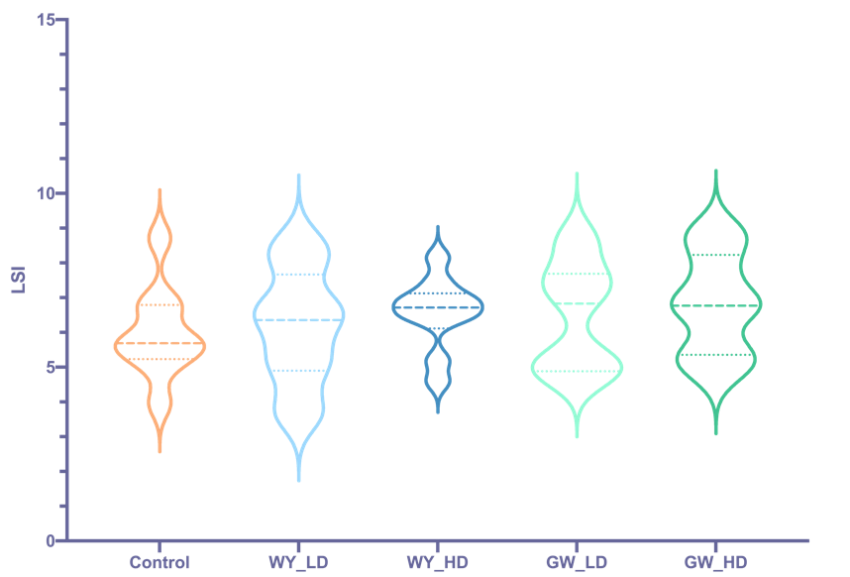


**Figure S1: Liver somatic index (LSI) in male Atlantic cod showed no significant changes between Control, WY-14,643 low dose and high dose (WY_LD and WY_HD, respectively), and GW501516 low dose and high dose (GW_LD and GW_HD, respectively).** Results shown in a violin plot, with median and quartiles indicated, produced in GraphPad PRISM v. 9.


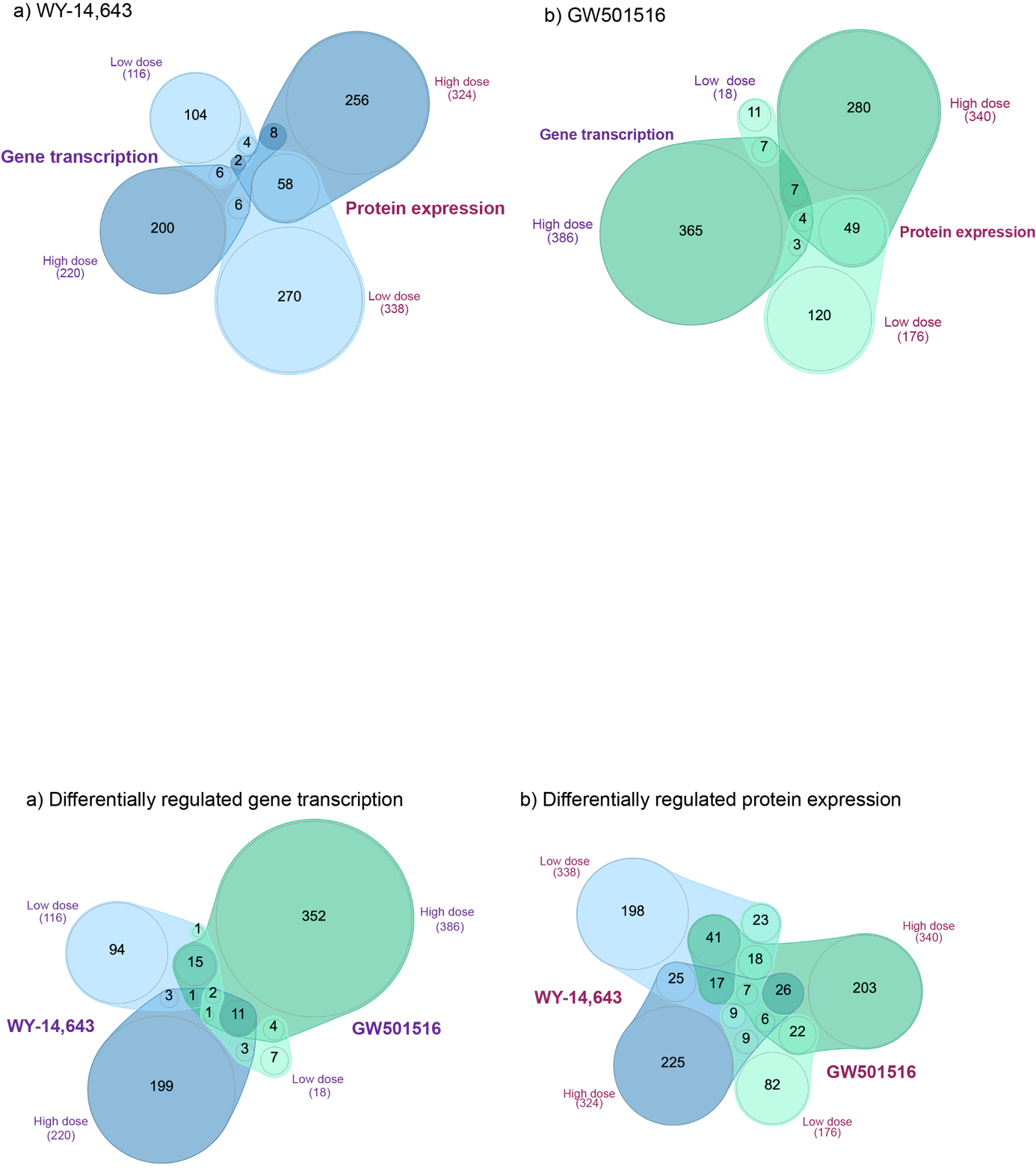


**Figure S2: Proportional overlap of differentially expressed a) genes (FDR < 0.05) and b) proteins (*p* < 0.05) in Atlantic cod liver following injections of WY-14,643 and GW501516**. Quasi-proportional Venn diagram was drawn using the nVenn tool (Perez-Silva et al. 2018). Note that for the lists used in pathway analysis, additional cutoff values of at least 1.5 and 1.2 fold-changes were applied to DEGs (Table S1A-C) and DEPs (Table S2A-C), respectively.


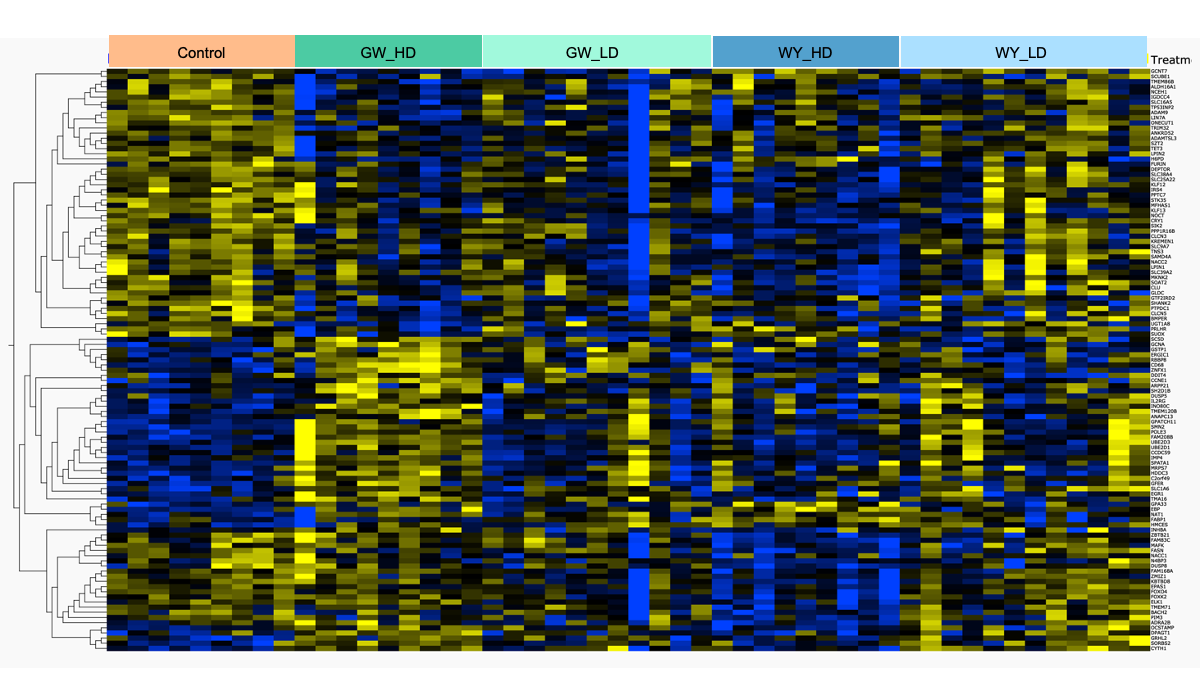


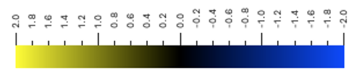


**Figure S3: One-way hierarchical clustering analysis of differentially expressed genes in liver of control group, GW501516 high (GW_HD, 4.0 mg/kg) and low (GW_LD, 0.4 mg/kg) dose, and WY-14,643 high (WY_HD, 40 mg/kg) and low (WY_LD, 4.0 mg/kg) dose treated Atlantic cod.** Analysis was performed based on log2-transformed ratio (treated/control) values of differentially expressed genes (FDR<0.125, FC > 1.5) in Multi Group Comparison (Qlucore Omics Explorer). The color bar at the bottom shows relative expression ranging from deep yellow (highest) to deep blue (lowest) levels. Rows represent genes and columns represent samples.


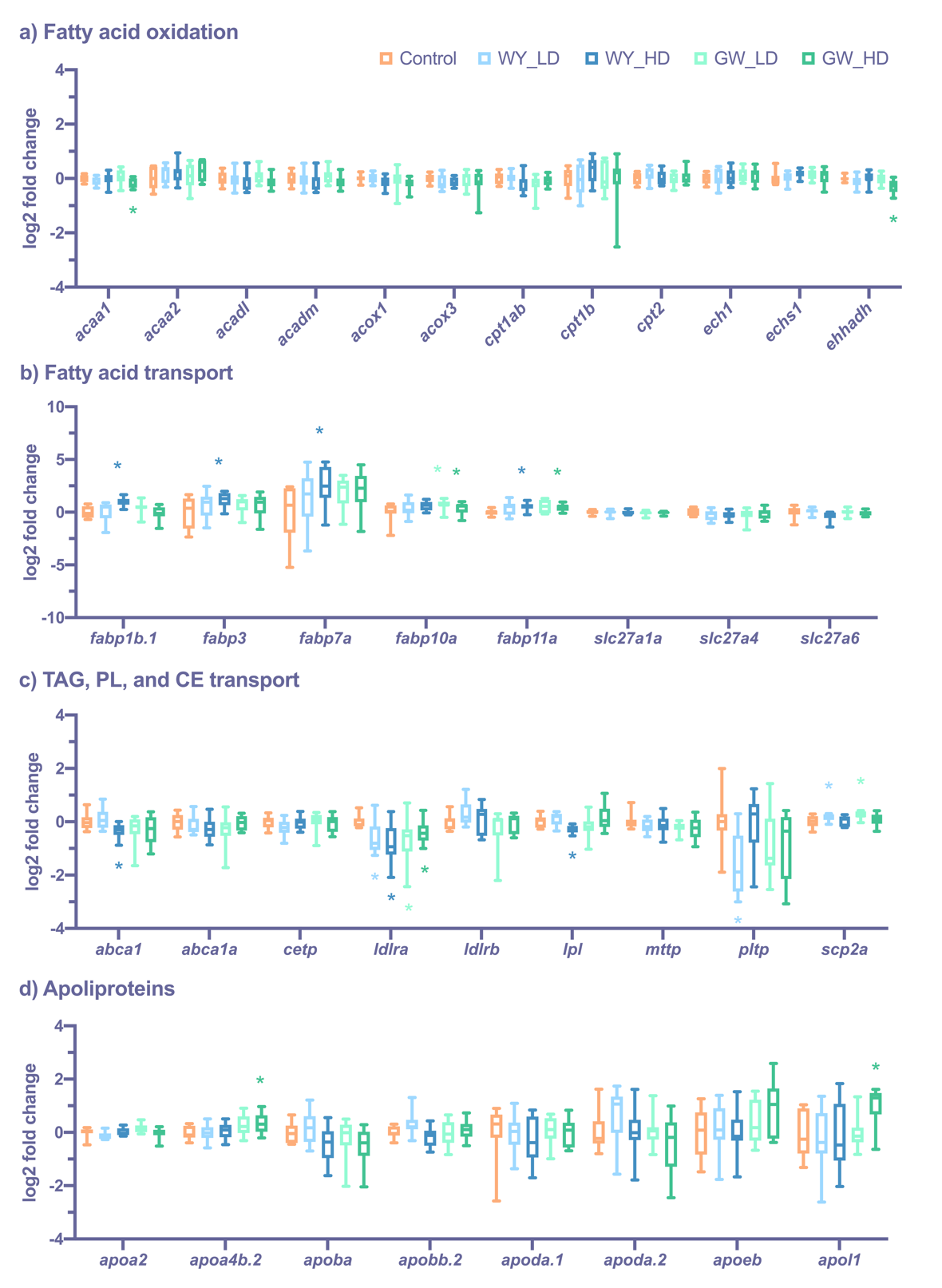


**Figure S4: Transcriptional effects on peroxisome proliferator-activated receptor (PPAR) target genes and processes from treatment with solvent control, WY-14,643 low and high dose (WY_LD and WY_HD; respectively), and GW501516 low and high dose (GW_LD and GW_HD, respectively).** PPAR target genes and their role was based on the KEGG “PPAR Signaling” pathway and literature, and is here grouped under a) Fatty acid oxidation, b) Fatty acid transport, c) Triglycerid (TAG), phospholipid (PL), and cholesterol ester (CE transportation, and d) Apolipoproteins. Results are shown as box plots with log2 transformed fold changes compared to control. Statistically significant changes (p < 0.05) were found using two-sample t-tests and are indicated with asterisk.

**Figure S5: Volcano plots of lipid species in cod liver microsomes following treatment with a) WY-14,643 high dose and b) GW501516 high dose compared to control.** The levels of lipids were measured using targeted FIA-HRMS lipidomics. The lipids that were statistically significant (p < 0.05 and fold change >1.5) affected by the high dose exposure of the compounds are colored red. The group of triglycerides (TGs) are circled.

**Figure S6: Volcano plots of lipid species in cod plasma following treatment with a) WY-14,643 high dose and b) GW501516 high dose.** The levels of lipids were measured using targeted FIA-HRMS lipidomics. The lipids that were statistically significant (p < 0.05 and fold change >1.5) affected by the high dose exposure of the compounds are colored red. The group of triglycerides (TGs) are circled.

**
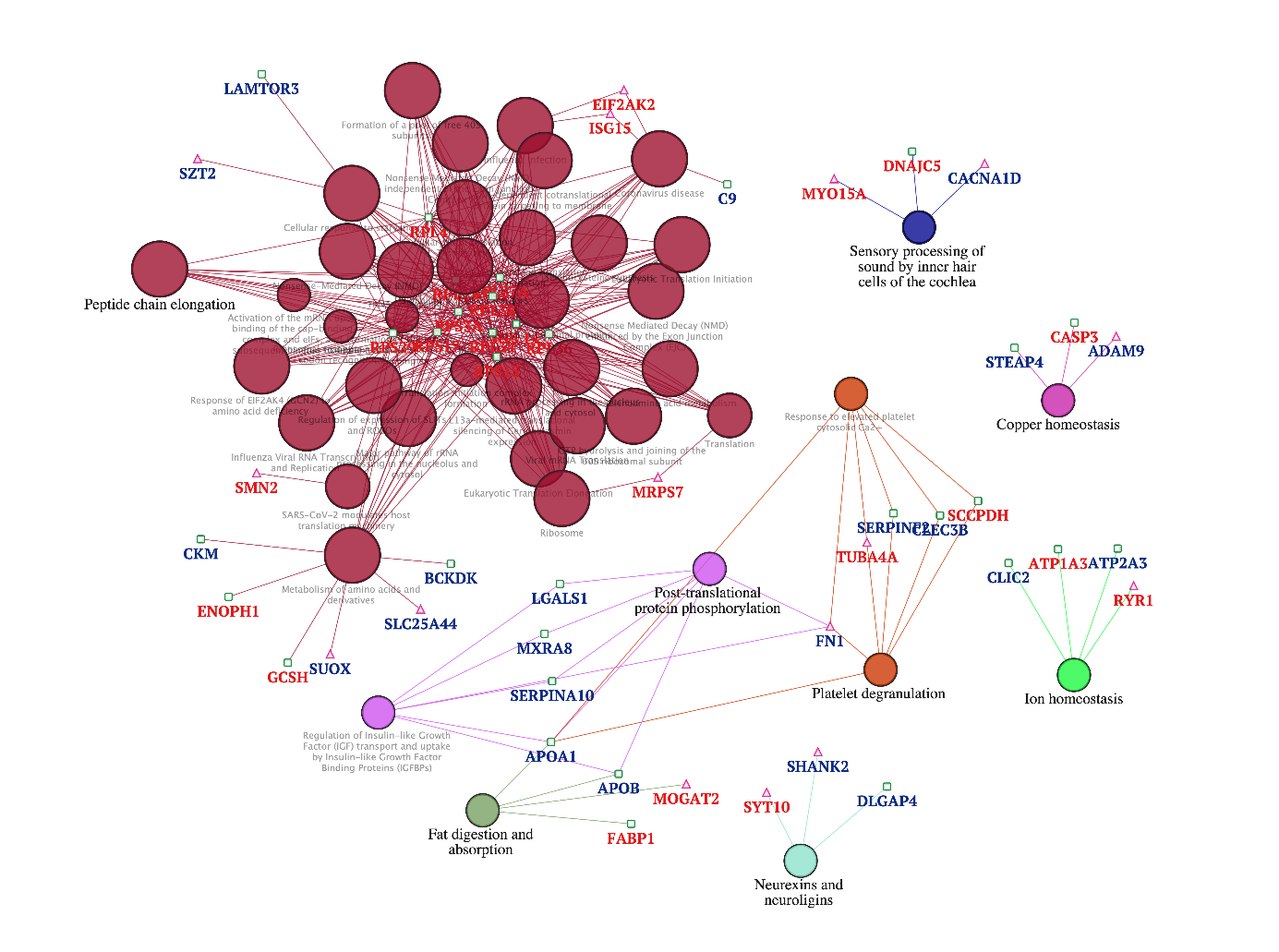
**

**Figure S7:** **Cytoscape networks showing enriched pathways in merged list of DEGs and DEPs from cod treated with GW501516.** Significantly enriched networks (adjusted *p*-value <0.05) generated using ClueGo with Wikipathway, KEGG and Reactome databases are shown. Gene/protein symbols shown in red and blue indicate up-regulation and down-regulation, respectively. Triangle, square and diamond symbols, represent transcripts, proteins and both (transcripts and proteins), respectively.

**2 Supplementary Tables**

**Table S1. Composition and ingredients of the feed used in the study (Amber Neptune, Skretting, Stavanger, Norway, batch no. 3343368).**

| **Parameter*** | **Content** |
| --- | --- |
| Protein % | 54-57 |
| Fat % | 15 |
| Nitrogen-free extracts % | 9-11 |
| Fiber % | 0.6-4.5 |
| Ash % | 10-12 |
| Gross energy MJ/kg | 20.5-21.5 |
| Pigment mg | - |
| Pellet size mm | 4 |

*Ingredients: Fish meal, wheat gluten, wheat, fish oil, faba beans

**Table S2A-D. Differentially expressed genes (DEGs) (FDR < 0.05 and fold-change (FC) ≥ 1.5) in Atlantic cod liver treated with low and high doses of WY-14,643 and GW501516.**

**Table S2A. DEGs from low dose of WY-14,643 treatment**

| Atlantic cod gene ID | Zebrafish gene/protein | Human gene/protein | FC | FDR |
| --- | --- | --- | --- | --- |
| ENSGMOG00000020289 |  |  | 0,13 | 7,04E-07 |
| ENSGMOG00000013370 | si:ch73-233k15.2 | APOL1 | 0,15 | 2,25E-05 |
| ENSGMOG00000004325 | colq | COLQ | 5,80 | 8,51E-05 |
| ENSGMOG00000010596 | aldoca | ALDOC | 0,44 | 8,82E-05 |
| ENSGMOG00000019814 |  |  | 171,84 | 1,15E-04 |
| ENSGMOG00000009507 | si:ch73-78o10.1 | DTX3L | 3,29 | 7,54E-04 |
| ENSGMOG00000014403 | havcr1 | TIMD4 | 0,23 | 3,34E-03 |
| ENSGMOG00000019868 |  |  | 4,21 | 3,34E-03 |
| ENSGMOG00000011895 | map6d1 | MAP6D1 | 0,27 | 3,34E-03 |
| ENSGMOG00000001639 | CU914164.3 | TLR8 | 7,00 | 3,80E-03 |
| ENSGMOG00000009261 | sox9b | SOX9 | 1,50 | 3,95E-03 |
| ENSGMOG00000001689 | CU914164.3 | TLR8 | 4,53 | 3,95E-03 |
| ENSGMOG00000013177 | rpl31 | RPL31 | 0,57 | 4,39E-03 |
| ENSGMOG00000009453 |  |  | 0,66 | 8,76E-03 |
| ENSGMOG00000008651 | nipal4 | NIPAL4 | 0,14 | 9,80E-03 |
| ENSGMOG00000005338 | xpnpep2 | XPNPEP2 | 3,14 | 9,80E-03 |
| ENSGMOG00000011234 | ppp1r14aa | PPP1R14A | 0,23 | 1,09E-02 |
| ENSGMOG00000016609 | cecr5 | HDHD5 | 0,51 | 1,09E-02 |
| ENSGMOG00000011085 | zap70 | ZAP70 | 1,98 | 1,21E-02 |
| ENSGMOG00000016171 |  |  | 2,21 | 1,21E-02 |
| ENSGMOG00000020555 |  |  | 2,29 | 1,21E-02 |
| ENSGMOG00000012381 | fstl5 | FSTL5 | 5,91 | 1,28E-02 |
| ENSGMOG00000006717 | slc1a6 | SLC1A6 | 5,91 | 1,28E-02 |
| ENSGMOG00000002179 | LGALS3BP | LGALS3BP | 0,46 | 1,32E-02 |
| ENSGMOG00000008868 | si:ch211-165b19.8 | GZMB | 2,20 | 1,33E-02 |
| ENSGMOG00000001612 | sepw2a | MIEN1 | 0,56 | 1,39E-02 |
| ENSGMOG00000005970 |  |  | 2,34 | 1,55E-02 |
| ENSGMOG00000012172 | mogat2 | MOGAT2 | 4,37 | 1,56E-02 |
| ENSGMOG00000000097 | lect2l | LECT2 | 0,35 | 1,86E-02 |
| ENSGMOG00000014363 | havcr1 | TIMD4 | 0,25 | 2,44E-02 |
| ENSGMOG00000012777 | pamr1 | PAMR1 | 0,55 | 2,46E-02 |
| ENSGMOG00000019781 | mab21l2 | MAB21L2 | 0,46 | 2,54E-02 |
| ENSGMOG00000012746 | havcr1 | TIMD4 | 0,46 | 2,61E-02 |
| ENSGMOG00000013968 | serpinb1l2 | SERPINB9 | 1,53 | 2,65E-02 |
| ENSGMOG00000009941 | slc1a4 | SLC1A4 | 0,22 | 2,65E-02 |
| ENSGMOG00000014718 |  |  | 70,15 | 2,84E-02 |
| ENSGMOG00000013096 | zgc:162879 |  | 2,98 | 2,99E-02 |
| ENSGMOG00000001909 | pycr1b | AL117348.2 | 0,60 | 3,07E-02 |
| ENSGMOG00000000377 | ddit4 | DDIT4 | 0,53 | 3,24E-02 |
| ENSGMOG00000010216 | ar | AR | 0,61 | 3,35E-02 |
| ENSGMOG00000000077 | aclya | ACLY | 8,70 | 3,36E-02 |
| ENSGMOG00000015708 | her6 | HES1 | 1,54 | 3,59E-02 |
| ENSGMOG00000006403 | FAM46A | FAM46A | 0,38 | 3,59E-02 |
| ENSGMOG00000010395 | tfa | TF | 0,63 | 3,70E-02 |
| ENSGMOG00000020165 |  |  | 2,03 | 3,70E-02 |
| ENSGMOG00000013167 |  | G0S2 | 1,86 | 3,70E-02 |
| ENSGMOG00000009563 | cyth4a | CYTH4 | 1,56 | 3,70E-02 |
| ENSGMOG00000012858 |  |  | 3,37 | 3,70E-02 |
| ENSGMOG00000000968 | CU651662.1 |  | 5,29 | 3,70E-02 |
| ENSGMOG00000010944 | cks1b | CKS1B | 0,62 | 3,74E-02 |
| ENSGMOG00000005403 | si:ch73-252i11.1 |  | 2,19 | 3,78E-02 |
| ENSGMOG00000017659 | rasal3 | RASAL3 | 1,54 | 3,78E-02 |
| ENSGMOG00000020467 | arl4cb | ARL4C | 1,57 | 3,82E-02 |
| ENSGMOG00000007116 | ccdc80 | CCDC80 | 1,60 | 3,82E-02 |
| ENSGMOG00000010578 | COLGALT1 | COLGALT1 | 0,51 | 3,82E-02 |
| ENSGMOG00000010286 | mpx |  | 1,93 | 3,82E-02 |
| ENSGMOG00000014164 | si:ch73-335l21.4 |  | 0,31 | 3,82E-02 |
| ENSGMOG00000007888 |  | CPZ | 0,64 | 3,82E-02 |
| ENSGMOG00000010751 | plaub | PLAU | 1,76 | 3,82E-02 |
| ENSGMOG00000009788 | aim1a | CRYBG1 | 1,57 | 3,82E-02 |
| ENSGMOG00000010880 | ptgs2a | PTGS2 | 1,57 | 3,87E-02 |
| ENSGMOG00000019144 | slc25a48 | SLC25A48 | 0,60 | 4,16E-02 |
| ENSGMOG00000001968 | tnfrsf11b |  | 0,27 | 4,22E-02 |
| ENSGMOG00000010566 |  |  | 1,55 | 4,22E-02 |
| ENSGMOG00000014045 | ehf | EHF | 1,64 | 4,22E-02 |
| ENSGMOG00000008724 | spegb | SPEG | 6,71 | 4,40E-02 |
| ENSGMOG00000006587 |  |  | 0,23 | 4,86E-02 |
| ENSGMOG00000016019 | cacna1sa | CACNA1S | 13,35 | 4,88E-02 |
| ENSGMOG00000006431 | pltp | PLTP | 0,33 | 4,88E-02 |
| ENSGMOG00000014112 | slc4a4a | SLC4A4 | 1,57 | 4,88E-02 |
| ENSGMOG00000019492 | CYP46A1 | CYP46A1 | 2,27 | 4,90E-02 |
| ENSGMOG00000016531 | si:dkey-286j15.3 |  | 1,69 | 4,92E-02 |

**Table S2B. DEGs from high dose of WY-14,643 treatment**

| Atlantic cod gene ID | Zebrafish gene/protein | Human gene/protein | FC | FDR |
| --- | --- | --- | --- | --- |
| ENSGMOG00000002059 | klf13 | KLF13 | 0,43 | 2,02E-09 |
| ENSGMOG00000006796 | ebp | EBP | 2,00 | 6,36E-06 |
| ENSGMOG00000013032 | | TNS3 | 0,53 | 3,21E-05 |
| ENSGMOG00000007929 | ppp1r16b | PPP1R16B | 0,61 | 3,21E-05 |
| ENSGMOG00000001619 | LPIN1 | LPIN1 | 0,42 | 4,33E-05 |
| ENSGMOG00000012189 | zbtb21 | ZBTB21 | 0,52 | 1,76E-04 |
| ENSGMOG00000012270 | nocta | NOCT | 0,27 | 1,76E-04 |
| ENSGMOG00000005291 | lipin2 | LPIN2 | 0,55 | 1,76E-04 |
| ENSGMOG00000001088 | cry1aa | CRY1 | 0,59 | 4,30E-04 |
| ENSGMOG00000000779 | rorca |  | 0,62 | 1,68E-03 |
| ENSGMOG00000005697 | fasn | FASN | 0,54 | 1,84E-03 |
| ENSGMOG00000008490 | acot13 | ACOT13 | 1,55 | 2,24E-03 |
| ENSGMOG00000019561 | klf12b | KLF12 | 0,67 | 2,33E-03 |
| ENSGMOG00000003279 | sik1 | CU639417.2 | 0,25 | 2,67E-03 |
| ENSGMOG00000014970 | col4a3bpb | COL4A3BP | 0,61 | 3,57E-03 |
| ENSGMOG00000007554 | fabp1b.1 | FABP1 | 2,04 | 3,57E-03 |
| ENSGMOG00000011606 | gpa33 | GPA33 | 1,93 | 3,57E-03 |
| ENSGMOG00000001689 | CU914164.3 | TLR8 | 5,52 | 3,60E-03 |
| ENSGMOG00000000277 | zmiz1a | ZMIZ1 | 0,62 | 3,60E-03 |
| ENSGMOG00000014331 | tor3a | TOR3A | 3,16 | 3,67E-03 |
| ENSGMOG00000003558 | fam83c | FAM83C | 0,56 | 3,81E-03 |
| ENSGMOG00000014960 | stk35l | STK35 | 0,62 | 3,81E-03 |
| ENSGMOG00000002370 | |  | 0,60 | 3,81E-03 |
| ENSGMOG00000016648 | DTX4 | DTX4 | 0,63 | 3,88E-03 |
| ENSGMOG00000017408 | cry5 |  | 2,07 | 4,45E-03 |
| ENSGMOG00000019814 | |  | 55,27 | 4,68E-03 |
| ENSGMOG00000013781 | klf15 |  | 0,38 | 4,99E-03 |
| ENSGMOG00000013437 | | RNF19A | 0,62 | 5,04E-03 |
| ENSGMOG00000017510 | si:ch211-235o23.1 | TMEM71 | 0,65 | 5,04E-03 |
| ENSGMOG00000008491 | sik2b | SIK2 | 0,61 | 5,04E-03 |
| ENSGMOG00000003686 | |  | 0,58 | 5,05E-03 |
| ENSGMOG00000012835 | | IRS4 | 0,46 | 5,13E-03 |
| ENSGMOG00000007497 | CABZ01049925.1 | | 1,89 | 5,13E-03 |
| ENSGMOG00000002885 | foxo4 | FOXO4 | 0,63 | 5,13E-03 |
| ENSGMOG00000017094 | gadd45aa | GADD45A | 2,01 | 5,43E-03 |
| ENSGMOG00000010970 | samd4a | SAMD4A | 0,60 | 5,87E-03 |
| ENSGMOG00000012486 | ccdc149b | CCDC149 | 1,58 | 6,08E-03 |
| ENSGMOG00000020296 | | DIO3 | 0,29 | 6,38E-03 |
| ENSGMOG00000004657 | slc9a7 | SLC9A7 | 0,53 | 7,27E-03 |
| ENSGMOG00000013720 | tnc | TNC | 4,99 | 7,27E-03 |
| ENSGMOG00000009172 | c9 | C9 | 2,02 | 7,54E-03 |
| ENSGMOG00000008393 | ero1a | ERO1A | 1,96 | 7,54E-03 |
| ENSGMOG00000006840 | n4bp3 | N4BP3 | 0,58 | 7,65E-03 |
| ENSGMOG00000012172 | mogat2 | MOGAT2 | 4,49 | 7,76E-03 |
| ENSGMOG00000019550 | | NOCT | 0,56 | 7,94E-03 |
| ENSGMOG00000009808 | bach2b | BACH2 | 0,50 | 8,14E-03 |
| ENSGMOG00000013011 | igfbp1b | IGFBP1 | 0,22 | 8,30E-03 |
| ENSGMOG00000019482 | ppib | PPIB | 1,57 | 9,70E-03 |
| ENSGMOG00000015021 | tmem56b |  | 0,65 | 1,03E-02 |
| ENSGMOG00000000377 | ddit4 | DDIT4 | 0,51 | 1,07E-02 |
| ENSGMOG00000010919 | peli1b | PELI1 | 0,30 | 1,07E-02 |
| ENSGMOG00000017783 | | NR3C1 | 0,64 | 1,07E-02 |
| ENSGMOG00000010170 | pptc7b | PPTC7 | 0,65 | 1,07E-02 |
| ENSGMOG00000001605 | foxk2 | FOXK2 | 0,66 | 1,07E-02 |
| ENSGMOG00000013300 | lmna | LMNA | 3,84 | 1,07E-02 |
| ENSGMOG00000019298 | slc25a25a | SLC25A25 | 0,25 | 1,07E-02 |
| ENSGMOG00000019180 | nr1d2a | NR1D2 | 0,56 | 1,08E-02 |
| ENSGMOG00000005910 | filip1l | FILIP1L | 0,37 | 1,09E-02 |
| ENSGMOG00000011105 | mknk2b | MKNK2 | 0,51 | 1,11E-02 |
| ENSGMOG00000011314 | hmces | HMCES | 1,53 | 1,17E-02 |
| ENSGMOG00000005621 | |  | 0,62 | 1,18E-02 |
| ENSGMOG00000006738 | crb2a | CRB2 | 0,27 | 1,22E-02 |
| ENSGMOG00000013429 | CU570791.1 |  | 4,08 | 1,23E-02 |
| ENSGMOG00000001247 | tpte | TPTE2 | 2,05 | 1,32E-02 |
| ENSGMOG00000017339 | slc39a1 | SLC39A2 | 0,64 | 1,53E-02 |
| ENSGMOG00000011939 | bsk146 |  | 0,51 | 1,59E-02 |
| ENSGMOG00000011938 | fabp11a | FABP12 | 1,51 | 1,66E-02 |
| ENSGMOG00000001227 | soat2 | SOAT2 | 0,47 | 1,79E-02 |
| ENSGMOG00000006739 | rapgef5a | RAPGEF5 | 0,61 | 1,82E-02 |
| ENSGMOG00000014203 | soat1 | SOAT1 | 0,46 | 1,89E-02 |
| ENSGMOG00000014975 | nr2f5 |  | 0,41 | 1,98E-02 |
| ENSGMOG00000016425 | lima1a | LIMA1 | 1,57 | 2,11E-02 |
| ENSGMOG00000007129 | ttbk2a | TTBK2 | 0,66 | 2,15E-02 |
| ENSGMOG00000003215 | GLDC | GLDC | 0,58 | 2,15E-02 |
| ENSGMOG00000004123 | | NACC2 | 0,62 | 2,20E-02 |
| ENSGMOG00000008358 | map2k1 | MAP2K1 | 3,06 | 2,32E-02 |
| ENSGMOG00000001841 | camk1b | CAMK1 | 0,66 | 2,40E-02 |
| ENSGMOG00000019255 | alox5ap | ALOX5AP | 2,72 | 2,40E-02 |
| ENSGMOG00000007702 | c7a | C7 | 1,71 | 2,40E-02 |
| ENSGMOG00000020107 | zgc:101040 | NAT1 | 1,52 | 2,41E-02 |
| ENSGMOG00000001757 | fen1 | FEN1 | 1,59 | 2,42E-02 |
| ENSGMOG00000007996 | ddit3 | DDIT3 | 0,62 | 2,42E-02 |
| ENSGMOG00000013707 | deptor | DEPTOR | 0,59 | 2,44E-02 |
| ENSGMOG00000017142 | fgfrl1a | FGFRL1 | 0,65 | 2,51E-02 |
| ENSGMOG00000003140 | enc1 | ENC1 | 0,52 | 2,56E-02 |
| ENSGMOG00000001267 | gpx7 | GPX7 | 2,23 | 2,69E-02 |
| ENSGMOG00000018187 | si:dkey-76k16.5 | GLYAT | 1,58 | 2,74E-02 |
| ENSGMOG00000012984 | mfhas1 | MFHAS1 | 0,60 | 2,74E-02 |
| ENSGMOG00000004404 | rpl31 | RPL31 | 1,58 | 2,74E-02 |
| ENSGMOG00000007674 | acsf2 | ACSF2 | 1,52 | 2,74E-02 |
| ENSGMOG00000001478 | CABZ01078737.1 | IL1R2 | 0,15 | 2,74E-02 |
| ENSGMOG00000016915 | lsp1 | LSP1 | 0,64 | 2,74E-02 |
| ENSGMOG00000000942 | |  | 1,95 | 2,79E-02 |
| ENSGMOG00000012274 | | VMP1 | 0,50 | 2,79E-02 |
| ENSGMOG00000011132 | zgc:77287 | LRR1 | 1,52 | 2,84E-02 |
| ENSGMOG00000008524 | elk1 | ELK1 | 0,66 | 2,95E-02 |
| ENSGMOG00000000089 | gria3a | GRIA3 | 0,52 | 3,01E-02 |
| ENSGMOG00000000022 | tph1b | TPH1 | 3,09 | 3,15E-02 |
| ENSGMOG00000001264 | mafk | MAFK | 0,57 | 3,35E-02 |
| ENSGMOG00000016785 | sh3gl3a | SH3GL3 | 0,65 | 3,40E-02 |
| ENSGMOG00000000613 | PDGFD | PDGFD | 0,54 | 3,69E-02 |
| ENSGMOG00000006844 | sytl1 | SYTL1 | 1,57 | 3,73E-02 |
| ENSGMOG00000004690 | anxa3b | ANXA3 | 1,58 | 3,81E-02 |
| ENSGMOG00000003625 | slc38a4 | SLC38A4 | 0,61 | 3,81E-02 |
| ENSGMOG00000011144 | klf11b | KLF11 | 2,50 | 4,00E-02 |
| ENSGMOG00000008325 | pacs2 | PACS2 | 0,66 | 4,19E-02 |
| ENSGMOG00000008588 | ercc5 | ERCC5 | 1,51 | 4,19E-02 |
| ENSGMOG00000014515 | si:ch1073-456m8.1 | | 0,66 | 4,19E-02 |
| ENSGMOG00000018924 | clu | CLU | 0,64 | 4,19E-02 |
| ENSGMOG00000010154 | |  | 0,53 | 4,66E-02 |
| ENSGMOG00000014865 | zgc:153952 | STK17A | 0,67 | 4,66E-02 |
| ENSGMOG00000007822 | ZSWIM6 | ZSWIM6 | 0,57 | 4,83E-02 |
| ENSGMOG00000012703 | MYO1D | MYO1D | 0,64 | 4,86E-02 |
| ENSGMOG00000015717 | rpa2 | RPA2 | 1,50 | 4,86E-02 |
| ENSGMOG00000013752 | igdcc4 | IGDCC4 | 0,62 | 4,89E-02 |
| ENSGMOG00000009265 | kremen1 | KREMEN1 | 0,66 | 4,89E-02 |
| ENSGMOG00000015403 | | PZP | 1,58 | 4,89E-02 |
| ENSGMOG00000008272 | fkbp7 | FKBP7 | 2,39 | 4,89E-02 |
| ENSGMOG00000005117 | bag2 | BAG2 | 2,03 | 4,89E-02 |
| ENSGMOG00000001493 | tle3b | TLE3 | 0,57 | 4,89E-02 |
| ENSGMOG00000017191 | cdh4 | CDH4 | 0,45 | 4,89E-02 |
| ENSGMOG00000017938 | mafbb | MAFB | 1,85 | 4,89E-02 |
| ENSGMOG00000001165 | igf1 | IGF1 | 0,62 | 4,94E-02 |

**Table S2C. DEGs from low dose of GW501516 treatment**

| Atlantic cod gene ID | Zebrafish gene/protein | Human gene/protein | FC | FDR |
| --- | --- | --- | --- | --- |
| ENSGMOG00000017183 | spon2b | SPON2 | 0,09 | 3,36E-03 |
| ENSGMOG00000019814 | |  | 52,40 | 3,36E-03 |
| ENSGMOG00000003540 | |  | 1,91 | 3,36E-03 |
| ENSGMOG00000012270 | nocta | NOCT | 0,35 | 3,36E-03 |
| ENSGMOG00000003279 | sik1 | CU639417.2 | 0,24 | 3,36E-03 |
| ENSGMOG00000012172 | mogat2 | MOGAT2 | 6,27 | 2,08E-02 |
| ENSGMOG00000006587 | |  | 0,15 | 2,33E-02 |
| ENSGMOG00000009018 | si:dkey-222b8.4 | KIAA0895 | 1,56 | 2,86E-02 |
| ENSGMOG00000014960 | stk35l | STK35 | 0,65 | 2,99E-02 |

**Table S2D. DEGs from high dose of GW501516 treatment**

| Atlantic cod gene ID | Zebrafish gene/protein | Human gene/protein | FC | FDR |
| --- | --- | --- | --- | --- |
| ENSGMOG00000003540 |  |  | 3,05 | 2,77E-09 |
| ENSGMOG00000013752 | igdcc4 | IGDCC4 | 0,50 | 2,82E-06 |
| ENSGMOG00000003589 | magixa |  | 0,54 | 2,82E-06 |
| ENSGMOG00000015140 | nceh1b.1 | NCEH1 | 0,50 | 4,49E-06 |
| ENSGMOG00000000597 |  |  | 1,59 | 4,49E-06 |
| ENSGMOG00000020270 |  |  | 1,76 | 4,49E-06 |
| ENSGMOG00000009753 |  |  | 1,58 | 4,49E-06 |
| ENSGMOG00000007343 | ankrd52a | ANKRD52 | 0,65 | 5,57E-06 |
| ENSGMOG00000003503 | hddc3 | HDDC3 | 1,60 | 2,38E-05 |
| ENSGMOG00000014932 | syne1a | SYNE1 | 0,66 | 2,38E-05 |
| ENSGMOG00000009849 | clcn4 | CLCN4 | 0,65 | 3,71E-05 |
| ENSGMOG00000016374 |  | VPS13B | 0,58 | 5,82E-05 |
| ENSGMOG00000010146 | fam208b | FAM208B | 1,54 | 5,82E-05 |
| ENSGMOG00000006301 |  |  | 1,59 | 8,38E-05 |
| ENSGMOG00000019791 | znfx1 | ZNFX1 | 2,33 | 4,33E-04 |
| ENSGMOG00000002441 | HECTD4 | HECTD4 | 0,51 | 6,47E-04 |
| ENSGMOG00000018840 |  |  | 1,83 | 6,47E-04 |
| ENSGMOG00000000486 | fn1b | FN1 | 0,62 | 6,49E-04 |
| ENSGMOG00000018813 | eif2ak2 | EIF2AK2 | 1,58 | 6,60E-04 |
| ENSGMOG00000010145 | gngt1 | GNG11 | 0,06 | 6,60E-04 |
| ENSGMOG00000004889 |  |  | 1,64 | 7,25E-04 |
| ENSGMOG00000016304 | si:dkey-205k8.5 | SPATA1 | 1,70 | 7,63E-04 |
| ENSGMOG00000017426 |  |  | 1,68 | 9,03E-04 |
| ENSGMOG00000006884 |  | APOB | 1,61 | 9,18E-04 |
| ENSGMOG00000016731 | ank1b | ANK1 | 0,30 | 9,65E-04 |
| ENSGMOG00000015844 | onecut1 | ONECUT1 | 0,61 | 9,65E-04 |
| ENSGMOG00000003098 |  |  | 4,57 | 9,65E-04 |
| ENSGMOG00000007501 | ube2d2l | UBE2D3 | 1,63 | 1,09E-03 |
| ENSGMOG00000000625 | znfx1 | ZNFX1 | 2,16 | 1,18E-03 |
| ENSGMOG00000009863 | tmem120b | TMEM120B | 1,73 | 1,37E-03 |
| ENSGMOG00000017279 | smn1 | SMN2 | 1,65 | 1,37E-03 |
| ENSGMOG00000002493 | egr1 | EGR1 | 2,08 | 1,48E-03 |
| ENSGMOG00000020441 |  |  | 1,52 | 1,59E-03 |
| ENSGMOG00000020404 |  |  | 1,79 | 1,86E-03 |
| ENSGMOG00000006556 | nacc1b | NACC1 | 0,61 | 2,02E-03 |
| ENSGMOG00000016270 | dact2 | DACT2 | 0,45 | 2,28E-03 |
| ENSGMOG00000020170 | zbtb20 | ZBTB20 | 0,63 | 2,29E-03 |
| ENSGMOG00000005403 | si:ch73-252i11.1 | | 2,20 | 2,63E-03 |
| ENSGMOG00000000669 | znfx1 | ZNFX1 | 2,05 | 2,95E-03 |
| ENSGMOG00000007475 | TP53INP2 | TP53INP2 | 0,61 | 3,43E-03 |
| ENSGMOG00000011832 | hydin | HYDIN | 292,79 | 3,43E-03 |
| ENSGMOG00000014750 | ube2d2 |  | 1,55 | 3,43E-03 |
| ENSGMOG00000019512 | ocstamp | OCSTAMP | 1,58 | 3,45E-03 |
| ENSGMOG00000013464 | clcn5a | CLCN5 | 0,65 | 3,94E-03 |
| ENSGMOG00000008025 |  |  | 0,27 | 3,94E-03 |
| ENSGMOG00000016086 |  |  | 0,44 | 4,01E-03 |
| ENSGMOG00000014921 |  | SLC4A10 | 0,56 | 4,42E-03 |
| ENSGMOG00000007650 | SH2D1B | SH2D1B | 1,59 | 4,64E-03 |
| ENSGMOG00000016235 |  |  | 0,57 | 4,96E-03 |
| ENSGMOG00000016296 |  |  | 2,38 | 5,09E-03 |
| ENSGMOG00000004904 | ptpdc1a | PTPDC1 | 0,46 | 5,15E-03 |
| ENSGMOG00000018394 |  |  | 0,57 | 5,57E-03 |
| ENSGMOG00000014960 | stk35l | STK35 | 0,62 | 6,27E-03 |
| ENSGMOG00000016429 | h6pd | H6PD | 0,60 | 6,30E-03 |
| ENSGMOG00000020554 | si:dkey-188i13.10 | IFI6 | 23,00 | 6,96E-03 |
| ENSGMOG00000011571 | CABZ01077402.1 | MYRFL | 0,15 | 7,93E-03 |
| ENSGMOG00000005446 |  | TNFRSF11B | 0,55 | 7,93E-03 |
| ENSGMOG00000010566 |  |  | 1,67 | 8,03E-03 |
| ENSGMOG00000015991 | shank2 | SHANK2 | 0,43 | 8,05E-03 |
| ENSGMOG00000019180 | nr1d2a | NR1D2 | 0,63 | 8,05E-03 |
| ENSGMOG00000002009 | suox | SUOX | 0,56 | 8,05E-03 |
| ENSGMOG00000016393 |  | VPS13B | 0,64 | 8,05E-03 |
| ENSGMOG00000009826 |  |  | 2,34 | 8,05E-03 |
| ENSGMOG00000005260 |  | NFIB | 0,67 | 8,70E-03 |
| ENSGMOG00000003382 | fosl1b | FOSL1 | 10,34 | 8,80E-03 |
| ENSGMOG00000014634 | slc25a44a | SLC25A44 | 0,64 | 9,44E-03 |
| ENSGMOG00000010049 | KCNG2 | KCNG2 | 4,49 | 9,44E-03 |
| ENSGMOG00000008612 | CABZ01056637.1 | SZT2 | 0,65 | 1,13E-02 |
| ENSGMOG00000006566 | ergic1 | ERGIC1 | 2,07 | 1,16E-02 |
| ENSGMOG00000013516 |  |  | 1,86 | 1,26E-02 |
| ENSGMOG00000008079 | tet3 | TET3 | 0,62 | 1,39E-02 |
| ENSGMOG00000006516 |  |  | 1,84 | 1,42E-02 |
| ENSGMOG00000001072 | msmo1 | MSMO1 | 2,65 | 1,42E-02 |
| ENSGMOG00000019814 |  |  | 8,18 | 1,44E-02 |
| ENSGMOG00000016587 | zgc:153293 | CCDC34 | 1,85 | 1,44E-02 |
| ENSGMOG00000012098 |  | RASGEF1B | 2,51 | 1,44E-02 |
| ENSGMOG00000014951 |  |  | 1,54 | 1,47E-02 |
| ENSGMOG00000005734 | HTR3C | HTR3D | 2,66 | 1,55E-02 |
| ENSGMOG00000017830 | SLC25A22 | SLC25A22 | 0,51 | 1,57E-02 |
| ENSGMOG00000001881 |  | UGT1A8 | 0,41 | 1,58E-02 |
| ENSGMOG00000018514 | CT025887.1 |  | 48,41 | 1,59E-02 |
| ENSGMOG00000012172 | mogat2 | MOGAT2 | 5,54 | 1,59E-02 |
| ENSGMOG00000002306 | gpr34b | GPR34 | 0,48 | 1,74E-02 |
| ENSGMOG00000020198 | atpif1a | ATPIF1 | 1,64 | 1,74E-02 |
| ENSGMOG00000018346 | adamtsl3 | ADAMTSL3 | 0,65 | 1,74E-02 |
| ENSGMOG00000005235 |  |  | 0,59 | 1,80E-02 |
| ENSGMOG00000015282 | CABZ01085552.1 | | 0,54 | 1,81E-02 |
| ENSGMOG00000016837 | filip1b | FILIP1 | 0,65 | 1,88E-02 |
| ENSGMOG00000004658 | mrps7 | MRPS7 | 1,59 | 1,94E-02 |
| ENSGMOG00000006717 | slc1a6 | SLC1A6 | 3,07 | 1,94E-02 |
| ENSGMOG00000003222 | tlr9 | AC097637.1 | 2,66 | 1,94E-02 |
| ENSGMOG00000001409 | tma16 | TMA16 | 1,75 | 1,96E-02 |
| ENSGMOG00000005150 | anapc13 | ANAPC13 | 1,78 | 2,15E-02 |
| ENSGMOG00000000630 | znfx1 | ZNFX1 | 1,78 | 2,20E-02 |
| ENSGMOG00000000699 | furina | FURIN | 0,63 | 2,20E-02 |
| ENSGMOG00000010621 | myo15aa | MYO15A | 33,64 | 2,37E-02 |
| ENSGMOG00000008734 | si:ch211-202a12.4 | ISG15 | 1,88 | 2,54E-02 |
| ENSGMOG00000015912 | bmper | BMPER | 0,56 | 2,76E-02 |
| ENSGMOG00000020221 |  |  | 2,09 | 2,78E-02 |
| ENSGMOG00000019391 | acap3b | ACAP3 | 0,40 | 2,79E-02 |
| ENSGMOG00000009940 |  |  | 1,51 | 2,86E-02 |
| ENSGMOG00000012518 | gstp2 | GSTP1 | 1,60 | 2,87E-02 |
| ENSGMOG00000004477 | cygb1 | CYGB | 0,42 | 2,87E-02 |
| ENSGMOG00000011493 |  | PLD4 | 1,74 | 2,95E-02 |
| ENSGMOG00000018831 | gpatch11 | GPATCH11 | 1,56 | 3,00E-02 |
| ENSGMOG00000020292 | ggact.2 | GGACT | 0,65 | 3,00E-02 |
| ENSGMOG00000019332 | ryr1a | RYR1 | 9,03 | 3,08E-02 |
| ENSGMOG00000003317 | aldh16a1 | ALDH16A1 | 0,64 | 3,10E-02 |
| ENSGMOG00000008242 | si:dkeyp-9d4.2 | | 2,10 | 3,11E-02 |
| ENSGMOG00000019669 |  |  | 1,86 | 3,13E-02 |
| ENSGMOG00000010700 | gfer | GFER | 1,56 | 3,20E-02 |
| ENSGMOG00000017686 | CU984600.2 |  | 1,74 | 3,20E-02 |
| ENSGMOG00000017302 | zbtb22b | ZBTB22 | 0,65 | 3,23E-02 |
| ENSGMOG00000008458 | gramd2a | GRAMD2 | 0,66 | 3,26E-02 |
| ENSGMOG00000020610 |  |  | 6,30 | 3,32E-02 |
| ENSGMOG00000011997 | adam9 | ADAM9 | 0,64 | 3,32E-02 |
| ENSGMOG00000008141 | arid3b | ARID3B | 0,56 | 3,37E-02 |
| ENSGMOG00000006320 | acrc | GCNA | 1,74 | 3,54E-02 |
| ENSGMOG00000011802 | slc47a1 | SLC47A2 | 0,61 | 3,55E-02 |
| ENSGMOG00000000763 | rbbp8 | RBBP8 | 1,63 | 3,63E-02 |
| ENSGMOG00000002108 | thraa | THRA | 0,61 | 3,63E-02 |
| ENSGMOG00000002747 |  |  | 0,64 | 3,63E-02 |
| ENSGMOG00000003257 | syt10 | SYT10 | 13,03 | 3,69E-02 |
| ENSGMOG00000014153 |  | TMEM65 | 1,91 | 3,69E-02 |
| ENSGMOG00000005338 | xpnpep2 | XPNPEP2 | 2,71 | 3,70E-02 |
| ENSGMOG00000005375 | si:ch73-252i11.1 | | 1,78 | 3,70E-02 |
| ENSGMOG00000013050 |  |  | 2,22 | 3,73E-02 |
| ENSGMOG00000000646 |  |  | 0,66 | 3,78E-02 |
| ENSGMOG00000014162 | cacna1db | CACNA1D | 0,65 | 3,85E-02 |
| ENSGMOG00000012439 | si:ch211-134m17.9 | FAM35A | 0,64 | 3,85E-02 |
| ENSGMOG00000002192 | st3gal5 | ST3GAL5 | 0,63 | 3,92E-02 |
| ENSGMOG00000017593 |  |  | 2,36 | 3,92E-02 |
| ENSGMOG00000000097 | lect2l | LECT2 | 0,39 | 4,12E-02 |
| ENSGMOG00000013063 |  |  | 2,23 | 4,15E-02 |
| ENSGMOG00000013259 | tgm5l | TGM3 | 2,70 | 4,15E-02 |
| ENSGMOG00000008968 | npat | NPAT | 1,59 | 4,23E-02 |
| ENSGMOG00000014559 | tmem86b | TMEM86B | 0,49 | 4,25E-02 |
| ENSGMOG00000002176 | trim32 | TRIM32 | 0,66 | 4,33E-02 |
| ENSGMOG00000019247 | tuba8l2 | TUBA4A | 1,56 | 4,48E-02 |
| ENSGMOG00000004466 | HERC5 | HERC6 | 0,62 | 4,49E-02 |
| ENSGMOG00000006366 | pole3 | POLE3 | 1,55 | 4,52E-02 |
| ENSGMOG00000015790 | stxbp5l | STXBP5L | 30,39 | 4,53E-02 |
| ENSGMOG00000019762 |  |  | 1,63 | 4,53E-02 |
| ENSGMOG00000002993 | slc16a5a | SLC16A5 | 0,59 | 4,53E-02 |
| ENSGMOG00000019485 | ppiab |  | 1,54 | 4,53E-02 |
| ENSGMOG00000005243 |  |  | 0,65 | 4,88E-02 |
| ENSGMOG00000002566 |  | TOGARAM1 | 4,62 | 4,91E-02 |
| ENSGMOG00000020337 | si:dkey-188i13.10 | IFI6 | 5,75 | 4,92E-02 |
| ENSGMOG00000003487 | usp18 | USP41 | 1,91 | 4,95E-02 |

**Table S3A-D. Differentially expressed proteins (DEPs) (p < 0.05 and fold-change (FC) ≥ 1.5) in Atlantic cod liver treated with low and high doses of WY-14,643 and GW501516.**

**Table S3A. DEPs from low dose of WY-14,643 treatment**

| Atlantic cod gene ID | Zebrafish gene name/protein | Human gene/protein | FC | p-value |
| --- | --- | --- | --- | --- |
| ENSGMOG00000007243 | aacs | AACS | 1,54 | 1,19E-02 |
| ENSGMOG00000014237 | CABZ01067153.1 | TNPO2 | 1,54 | 3,72E-03 |
| ENSGMOG00000008163 | tmed1a | TMED1 | 1,50 | 1,56E-02 |
| ENSGMOG00000001931 | atp1a3a | ATP1A3 | 1,50 | 2,19E-02 |
| ENSGMOG00000000951 | sos2 | SOS2 | 1,47 | 8,64E-03 |
| ENSGMOG00000006910 | eapp | EAPP | 1,45 | 2,66E-02 |
| ENSGMOG00000014326 | ggh | GGH | 1,42 | 3,79E-02 |
| ENSGMOG00000016937 | lrp6 | LRP6 | 1,39 | 3,02E-02 |
| ENSGMOG00000019255 | alox5ap | ALOX5AP | 1,37 | 3,73E-02 |
| ENSGMOG00000006667 |  |  | 1,36 | 2,71E-02 |
| ENSGMOG00000012258 | sema4e |  | 1,35 | 3,08E-02 |
| ENSGMOG00000012032 | acacb | ACACB | 1,32 | 1,86E-02 |
| ENSGMOG00000008763 | RNF14 | RNF14 | 1,31 | 3,59E-02 |
| ENSGMOG00000015752 | cideb | CIDEB | 1,30 | 2,61E-02 |
| ENSGMOG00000010754 | rps18 | RPS18 | 1,29 | 5,67E-03 |
| ENSGMOG00000000077 | aclya | ACLY | 1,29 | 8,56E-03 |
| ENSGMOG00000013749 | pcna | PCNA | 1,29 | 4,29E-02 |
| ENSGMOG00000001741 | acaca | ACACA | 1,29 | 1,56E-02 |
| ENSGMOG00000011606 | gpa33 | GPA33 | 1,28 | 1,28E-02 |
| ENSGMOG00000012895 | c3a.6 | C3 | 1,28 | 4,25E-04 |
| ENSGMOG00000010471 | rps13 | RPS13 | 1,28 | 1,66E-02 |
| ENSGMOG00000015240 | fam98a | FAM98A | 1,28 | 1,70E-03 |
| ENSGMOG00000000653 | si:dkey-286j15.3 | | 1,25 | 2,96E-02 |
| ENSGMOG00000010686 | tecrb | TECR | 1,25 | 1,76E-03 |
| ENSGMOG00000005439 | asns | ASNS | 1,24 | 4,00E-03 |
| ENSGMOG00000013926 |  | DMTN | 1,24 | 2,94E-02 |
| ENSGMOG00000018492 | enoph1 | ENOPH1 | 1,24 | 3,33E-02 |
| ENSGMOG00000018776 | ube2d1a | UBE2D1 | 1,24 | 4,46E-02 |
| ENSGMOG00000016429 | h6pd | H6PD | 1,23 | 2,66E-02 |
| ENSGMOG00000011722 |  |  | 1,23 | 1,50E-02 |
| ENSGMOG00000016970 | gnpnat1 | GNPNAT1 | 1,23 | 4,14E-02 |
| ENSGMOG00000007069 | agpat2 | AGPAT2 | 1,22 | 4,99E-02 |
| ENSGMOG00000000624 | txndc5 | TXNDC5 | 1,21 | 6,70E-03 |
| ENSGMOG00000011191 | morc3a | MORC3 | 1,20 | 1,25E-02 |
| ENSGMOG00000020428 |  | IGKV4-1 | 0,81 | 1,97E-02 |
| ENSGMOG00000020362 | si:dkey-211g8.4 | | 0,81 | 1,07E-02 |
| ENSGMOG00000009295 |  | AIF1 | 0,81 | 2,64E-02 |
| ENSGMOG00000020449 | HIST2H2AB | HIST2H2AB | 0,81 | 4,90E-02 |
| ENSGMOG00000013837 | apoeb | APOE | 0,80 | 3,58E-03 |
| ENSGMOG00000002167 | irf9 | IRF9 | 0,80 | 4,93E-02 |
| ENSGMOG00000010811 | FO704622.1 |  | 0,80 | 1,49E-02 |
| ENSGMOG00000013853 | apoc1 |  | 0,79 | 2,96E-02 |
| ENSGMOG00000001652 |  |  | 0,79 | 4,61E-02 |
| ENSGMOG00000009793 |  |  | 0,79 | 4,70E-02 |
| ENSGMOG00000001124 | syk | SYK | 0,78 | 1,46E-02 |
| ENSGMOG00000013363 | sult3st1 |  | 0,78 | 4,24E-02 |
| ENSGMOG00000014574 |  |  | 0,74 | 3,35E-02 |
| ENSGMOG00000010771 | zgc:85789 | C11orf54 | 0,71 | 1,77E-02 |
| ENSGMOG00000002484 |  |  | 0,69 | 2,28E-03 |
| ENSGMOG00000015858 | si:ch211-214k5.6 | | 0,66 | 3,94E-02 |
| ENSGMOG00000004100 | apoa1b | APOA1 | 0,64 | 3,82E-02 |
| ENSGMOG00000010763 | aspa | ASPA | 0,63 | 1,06E-03 |
| ENSGMOG00000019686 | mxra8b | MXRA8 | 0,58 | 5,06E-03 |

**Table S3B. DEPs from high dose of WY-14,643 treatment**

| Atlantic cod gene ID | Zebrafish gene/protein | Human gene/protein | Fold-change | p-value |
| --- | --- | --- | --- | --- |
| ENSGMOG00000012286 | CR788316.4 |  | 2,62 | 3,26E-02 |
| ENSGMOG00000017552 | si:ch73-170d6.2 | | 1,88 | 4,90E-02 |
| ENSGMOG00000014326 | ggh | GGH | 1,51 | 4,01E-02 |
| ENSGMOG00000007554 | fabp1b.1 | FABP1 | 1,42 | 1,16E-02 |
| ENSGMOG00000003599 |  |  | 1,39 | 4,28E-02 |
| ENSGMOG00000005020 | gtf2e2 | GTF2E2 | 1,37 | 3,53E-02 |
| ENSGMOG00000002335 | ccdc50 | CCDC50 | 1,37 | 2,63E-02 |
| ENSGMOG00000017285 | dido1 | DIDO1 | 1,37 | 1,32E-02 |
| ENSGMOG00000000356 | tmeff2b | TMEFF2 | 1,36 | 6,24E-03 |
| ENSGMOG00000001931 | atp1a3a | ATP1A3 | 1,35 | 1,74E-02 |
| ENSGMOG00000012312 | sid4 | HMCN2 | 1,34 | 2,99E-04 |
| ENSGMOG00000009569 | si:dkey-51e6.1 | | 1,34 | 1,02E-02 |
| ENSGMOG00000015181 | ptpn11b |  | 1,32 | 4,27E-02 |
| ENSGMOG00000005166 | rpl38 | RPL38 | 1,30 | 3,44E-02 |
| ENSGMOG00000010247 | rps15 | RPS15 | 1,28 | 4,08E-02 |
| ENSGMOG00000009235 |  |  | 1,28 | 1,86E-03 |
| ENSGMOG00000010193 | glulb | GLUL | 1,27 | 7,63E-03 |
| ENSGMOG00000001303 | uchl1 | UCHL1 | 1,27 | 2,52E-02 |
| ENSGMOG00000007818 | pgm5 | PGM5 | 1,26 | 8,39E-03 |
| ENSGMOG00000015603 | yrk |  | 1,26 | 1,59E-02 |
| ENSGMOG00000018654 | fth1a | FTMT | 1,25 | 3,67E-02 |
| ENSGMOG00000004873 | dnajc5ab | DNAJC5 | 1,24 | 3,03E-03 |
| ENSGMOG00000003457 | vps33b | VPS33B | 1,24 | 2,09E-02 |
| ENSGMOG00000015173 | csrp1b | CSRP1 | 1,24 | 5,51E-03 |
| ENSGMOG00000017802 |  |  | 1,24 | 1,53E-03 |
| ENSGMOG00000010338 | chia.6 |  | 1,23 | 3,97E-03 |
| ENSGMOG00000019416 | fabp10a |  | 1,23 | 2,21E-02 |
| ENSGMOG00000013968 | serpinb1l2 | SERPINB9 | 1,23 | 2,16E-03 |
| ENSGMOG00000003353 | zgc:194125 |  | 1,22 | 2,51E-02 |
| ENSGMOG00000009441 | nudt9 | NUDT9 | 1,22 | 2,07E-02 |
| ENSGMOG00000000851 |  | RPS6KB2 | 1,22 | 1,43E-03 |
| ENSGMOG00000000082 | TBC1D9B | TBC1D9B | 1,22 | 4,52E-02 |
| ENSGMOG00000018038 | prkdc | PRKDC | 1,21 | 4,32E-03 |
| ENSGMOG00000015093 | crot | CROT | 1,21 | 4,64E-02 |
| ENSGMOG00000019405 | nfybb | NFYB | 1,21 | 3,45E-02 |
| ENSGMOG00000000482 | trim2a | TRIM2 | 0,80 | 2,58E-02 |
| ENSGMOG00000003571 | CT573860.1 |  | 0,80 | 3,53E-03 |
| ENSGMOG00000008734 | si:ch211-202a12.4 | ISG15 | 0,79 | 1,79E-02 |
| ENSGMOG00000000097 | lect2l | LECT2 | 0,79 | 5,79E-03 |
| ENSGMOG00000001652 |  |  | 0,78 | 6,72E-03 |
| ENSGMOG00000003804 | steap4 | STEAP4 | 0,77 | 2,00E-02 |
| ENSGMOG00000014574 |  |  | 0,76 | 1,11E-02 |
| ENSGMOG00000016282 | slc38a3b | SLC38A3 | 0,75 | 4,27E-02 |
| ENSGMOG00000014056 | prpf4 | PRPF4 | 0,75 | 1,59E-02 |
| ENSGMOG00000013280 | zgc:110329 |  | 0,75 | 2,73E-02 |
| ENSGMOG00000020428 |  | IGKV4-1 | 0,74 | 2,25E-03 |
| ENSGMOG00000012338 | atp2a3 | ATP2A3 | 0,74 | 1,31E-02 |
| ENSGMOG00000011496 | tegt | TMBIM6 | 0,71 | 6,90E-03 |
| ENSGMOG00000002000 |  | SAMHD1 | 0,70 | 4,77E-02 |
| ENSGMOG00000015997 | si:dkey-286j15.3 | | 0,69 | 3,48E-02 |
| ENSGMOG00000016728 | FP016018.1 | IGLC1 | 0,66 | 3,56E-03 |
| ENSGMOG00000008278 | trim35-40 | TRIM35 | 0,65 | 2,06E-02 |
| ENSGMOG00000016132 | tmem160 | TMEM160 | 0,65 | 9,95E-03 |
| ENSGMOG00000016297 | ywhag1 | YWHAG | 0,61 | 3,70E-02 |
| ENSGMOG00000019665 | iqsec2b | IQSEC2 | 0,58 | 3,03E-02 |

**Table S3C. DEPs from low dose of GW501516 treatment**

| Atlantic cod gene ID | Zebrafish gene/protein | Human gene/protein | Fold-change | p-value |
| --- | --- | --- | --- | --- |
| ENSGMOG00000008102 | c9 | C9 | 0,81 | 1,55E-03 |
| ENSGMOG00000017802 | |  | 1,23 | 1,70E-03 |
| ENSGMOG00000009605 | ckmb | CKM | 0,82 | 5,81E-03 |
| ENSGMOG00000002668 | gcshb | GCSH | 1,20 | 6,27E-03 |
| ENSGMOG00000018398 | pkp3a | PKP3 | 0,83 | 6,64E-03 |
| ENSGMOG00000018492 | enoph1 | ENOPH1 | 1,26 | 6,72E-03 |
| ENSGMOG00000006764 | hspb8 | HSPB8 | 1,21 | 1,04E-02 |
| ENSGMOG00000006741 | cptp | CPTP | 0,82 | 1,22E-02 |
| ENSGMOG00000004873 | dnajc5ab | DNAJC5 | 1,21 | 1,23E-02 |
| ENSGMOG00000003804 | steap4 | STEAP4 | 0,76 | 1,36E-02 |
| ENSGMOG00000000401 | steap4 | STEAP4 | 0,81 | 1,37E-02 |
| ENSGMOG00000015997 | si:dkey-286j15.3 | | 0,68 | 1,70E-02 |
| ENSGMOG00000009702 | rxrbb | RXRB | 0,82 | 1,73E-02 |
| ENSGMOG00000017830 | SLC25A22 | SLC25A22 | 0,82 | 1,93E-02 |
| ENSGMOG00000018874 | cacna2d1a | CACNA2D1 | 0,81 | 1,96E-02 |
| ENSGMOG00000006940 | armc8 | ARMC8 | 1,24 | 2,01E-02 |
| ENSGMOG00000009441 | nudt9 | NUDT9 | 1,30 | 2,07E-02 |
| ENSGMOG00000004023 | CABZ01038161.1 | LACTB2 | 0,80 | 2,28E-02 |
| ENSGMOG00000014429 | krt5 | KRT76 | 0,83 | 2,40E-02 |
| ENSGMOG00000014574 | |  | 0,72 | 2,92E-02 |
| ENSGMOG00000017286 | zgc:175088 | FBXO2 | 1,30 | 3,09E-02 |
| ENSGMOG00000006426 | |  | 1,20 | 3,52E-02 |
| ENSGMOG00000010827 | |  | 0,81 | 3,57E-02 |
| ENSGMOG00000003448 | gys2 | GYS2 | 0,83 | 3,71E-02 |
| ENSGMOG00000009295 | | AIF1 | 0,83 | 3,75E-02 |
| ENSGMOG00000019027 | becn1 | BECN2 | 1,22 | 4,20E-02 |
| ENSGMOG00000002927 | nol8 | NOL8 | 0,70 | 4,26E-02 |
| ENSGMOG00000006802 | zgc:66156 | MYH13 | 0,79 | 4,47E-02 |
| ENSGMOG00000010811 | FO704622.1 |  | 0,74 | 4,64E-02 |
| ENSGMOG00000016313 | myhz1.3 | MYH13 | 0,72 | 4,88E-02 |

**Table S3D. DEPs from high dose of GW501516 treatment**

| Atlantic cod gene ID | Zebrafish gene/protein | Human gene/protein | Fold-change | p-value |
| --- | --- | --- | --- | --- |
| ENSGMOG00000018789 | stx8 | STX8 | 0,49 | 7,61E-05 |
| ENSGMOG00000010390 | SERPINA10 | SERPINA10 | 0,83 | 1,64E-04 |
| ENSGMOG00000002484 |  |  | 0,69 | 6,77E-04 |
| ENSGMOG00000018398 | pkp3a | PKP3 | 0,78 | 2,47E-03 |
| ENSGMOG00000014521 |  | GIMAP1 | 0,77 | 2,57E-03 |
| ENSGMOG00000016590 | lgals2a | LGALS1 | 0,79 | 3,94E-03 |
| ENSGMOG00000009793 |  |  | 0,75 | 4,17E-03 |
| ENSGMOG00000009441 | nudt9 | NUDT9 | 1,28 | 4,21E-03 |
| ENSGMOG00000007554 | fabp1b.1 | FABP1 | 1,32 | 4,73E-03 |
| ENSGMOG00000000942 |  |  | 1,21 | 4,75E-03 |
| ENSGMOG00000010799 | rps29 | RPS29 | 1,23 | 5,27E-03 |
| ENSGMOG00000004100 | apoa1b | APOA1 | 0,74 | 5,52E-03 |
| ENSGMOG00000020497 |  | HBG1 | 0,65 | 5,55E-03 |
| ENSGMOG00000016717 | casp3b | CASP3 | 1,20 | 5,98E-03 |
| ENSGMOG00000013633 | sccpdha | SCCPDH | 1,24 | 6,13E-03 |
| ENSGMOG00000020266 | hbbe2 | HBG1 | 0,65 | 6,30E-03 |
| ENSGMOG00000010399 | serpinf2b | SERPINF2 | 0,82 | 6,86E-03 |
| ENSGMOG00000001640 | gorasp1b |  | 0,75 | 7,16E-03 |
| ENSGMOG00000015697 | cahz | CA1 | 0,77 | 8,00E-03 |
| ENSGMOG00000009605 | ckmb | CKM | 0,80 | 8,04E-03 |
| ENSGMOG00000005434 | clic2 | CLIC2 | 0,82 | 8,27E-03 |
| ENSGMOG00000011506 | aqp1a.1 | AC004691.2 | 0,77 | 8,42E-03 |
| ENSGMOG00000000871 | clec3ba | CLEC3B | 0,70 | 9,09E-03 |
| ENSGMOG00000012971 |  |  | 1,29 | 9,09E-03 |
| ENSGMOG00000015840 | hbbe2 | HBG1 | 0,68 | 9,14E-03 |
| ENSGMOG00000015971 |  |  | 0,79 | 9,36E-03 |
| ENSGMOG00000008102 | c9 | C9 | 0,81 | 9,54E-03 |
| ENSGMOG00000005197 |  | RPL27A | 1,35 | 9,98E-03 |
| ENSGMOG00000005709 | hbae1.3 |  | 0,61 | 1,10E-02 |
| ENSGMOG00000015858 | si:ch211-214k5.6 | | 0,71 | 1,16E-02 |
| ENSGMOG00000004057 | hbae1.3 |  | 0,65 | 1,19E-02 |
| ENSGMOG00000010247 | rps15 | RPS15 | 1,29 | 1,25E-02 |
| ENSGMOG00000004064 | pvalb4 |  | 0,76 | 1,35E-02 |
| ENSGMOG00000009714 | bckdk | BCKDK | 0,80 | 1,35E-02 |
| ENSGMOG00000002752 | rpl13 | RPL13 | 1,27 | 1,36E-02 |
| ENSGMOG00000011559 | tfdp1a | TFDP1 | 0,79 | 1,36E-02 |
| ENSGMOG00000016420 |  |  | 0,74 | 1,36E-02 |
| ENSGMOG00000000209 | pdzd8 | PDZD8 | 0,75 | 1,45E-02 |
| ENSGMOG00000007885 | me2 | ME2 | 0,82 | 1,51E-02 |
| ENSGMOG00000017188 | bloc1s3 | AC005779.2 | 0,83 | 1,52E-02 |
| ENSGMOG00000011496 | tegt | TMBIM6 | 0,74 | 1,52E-02 |
| ENSGMOG00000015384 | znf346 | ZNF346 | 0,76 | 1,60E-02 |
| ENSGMOG00000002000 |  | SAMHD1 | 0,67 | 1,75E-02 |
| ENSGMOG00000008359 | rpl4 | RPL4 | 1,30 | 1,75E-02 |
| ENSGMOG00000001567 | slc4a1a | SLC4A1 | 0,74 | 1,84E-02 |
| ENSGMOG00000019686 | mxra8b | MXRA8 | 0,79 | 1,88E-02 |
| ENSGMOG00000006042 | r3hdm1 | R3HDM1 | 0,80 | 1,89E-02 |
| ENSGMOG00000005861 | sat2b |  | 1,35 | 1,98E-02 |
| ENSGMOG00000015246 | mhc1uka | FCGRT | 1,21 | 2,05E-02 |
| ENSGMOG00000017733 | map3k5 | MAP3K5 | 0,63 | 2,18E-02 |
| ENSGMOG00000009796 | dlgap4a | DLGAP4 | 0,77 | 2,20E-02 |
| ENSGMOG00000006950 | ttc32 | TTC32 | 0,80 | 2,23E-02 |
| ENSGMOG00000012286 | CR788316.4 |  | 2,19 | 2,26E-02 |
| ENSGMOG00000014932 | syne1a | SYNE1 | 1,21 | 2,26E-02 |
| ENSGMOG00000000097 | lect2l | LECT2 | 0,82 | 2,27E-02 |
| ENSGMOG00000010881 | rpl36 | RPL36 | 1,51 | 2,34E-02 |
| ENSGMOG00000011090 | fabp7a | FABP7 | 1,23 | 2,39E-02 |
| ENSGMOG00000005166 | rpl38 | RPL38 | 1,32 | 2,50E-02 |
| ENSGMOG00000005117 | bag2 | BAG2 | 0,68 | 2,67E-02 |
| ENSGMOG00000003477 | rab6a | RAB6C | 0,80 | 2,79E-02 |
| ENSGMOG00000013363 | sult3st1 |  | 0,80 | 2,81E-02 |
| ENSGMOG00000005681 | psmb12 |  | 0,82 | 2,84E-02 |
| ENSGMOG00000015729 | ca2 | CA1 | 0,78 | 2,85E-02 |
| ENSGMOG00000008493 | rps17 | RPS17 | 1,48 | 2,91E-02 |
| ENSGMOG00000004935 | hdac10 | HDAC10 | 0,76 | 2,99E-02 |
| ENSGMOG00000017133 | apoba | APOB | 0,80 | 3,04E-02 |
| ENSGMOG00000018166 | sdr16c5a | SDR16C5 | 0,76 | 3,20E-02 |
| ENSGMOG00000014192 | rpl14 | RPL14 | 1,24 | 3,38E-02 |
| ENSGMOG00000003990 | pvalb2 |  | 0,74 | 3,46E-02 |
| ENSGMOG00000001931 | atp1a3a | ATP1A3 | 1,46 | 3,52E-02 |
| ENSGMOG00000009982 | rpl34 | RPL34 | 1,46 | 3,53E-02 |
| ENSGMOG00000001239 | fuom | FUOM | 1,69 | 3,65E-02 |
| ENSGMOG00000012461 | yipf1 | YIPF1 | 0,78 | 3,69E-02 |
| ENSGMOG00000001707 | rps3a | RPS3A | 1,27 | 3,74E-02 |
| ENSGMOG00000018874 | cacna2d1a | CACNA2D1 | 0,81 | 3,86E-02 |
| ENSGMOG00000002168 | vtna | VTN | 0,83 | 3,86E-02 |
| ENSGMOG00000019665 | iqsec2b | IQSEC2 | 0,62 | 3,89E-02 |
| ENSGMOG00000003571 | CT573860.1 |  | 0,83 | 3,91E-02 |
| ENSGMOG00000000916 | lamtor3 | LAMTOR3 | 0,80 | 3,94E-02 |
| ENSGMOG00000012338 | atp2a3 | ATP2A3 | 0,74 | 4,06E-02 |
| ENSGMOG00000016965 | bphl | BPHL | 0,78 | 4,15E-02 |
| ENSGMOG00000017830 | SLC25A22 | SLC25A22 | 0,71 | 4,26E-02 |
| ENSGMOG00000019084 | arhgap29a | ARHGAP29 | 0,72 | 4,29E-02 |
| ENSGMOG00000016282 | slc38a3b | SLC38A3 | 0,83 | 4,36E-02 |
| ENSGMOG00000013184 | myof | MYOF | 0,74 | 4,41E-02 |
| ENSGMOG00000015997 | si:dkey-286j15.3 | | 0,73 | 4,68E-02 |
| ENSGMOG00000017286 | zgc:175088 | FBXO2 | 1,28 | 4,69E-02 |

**Table S4. Identifiers of differentially abundant lipid compounds (DALs) (adjusted p < 0.05) from Atlantic cod liver treated with high dose of GW501516 (see Figure 4).**

| ID | Lipid compound (retention time_mass/charge) | Suggested lipid class^§^ | Log fold change | Adjusted p-value |
| --- | --- | --- | --- | --- |
| DAL 1 | 13.06_506.5292m/z | DG | -7,67E-04 | 7,93E-05 |
| DAL 2 | 2.06_282.2786m/z | MG | -5,96E-05 | 7,61E-04 |
| DAL 3 | 12.99_480.5111m/z | DG | -3,04E-05 | 3,04E-03 |
| DAL 4 | 13.67_508.5424m/z | DG | -3,15E-05 | 3,04E-03 |

^§^Zdenka Bartosova, personal communication. MG = monoglycerides, DG = diglycerides.

**Table S5. The top significantly enriched (FDR q value < 0.05) disease terms (DisGeNET datbase) in differentially expressed genes (p < 0.05) in liver of Atlantic cod exposed to low and high dose WY-14,643.**

| DisGeNET Name | q-value FDR B&H | Hit in Query List |
| --- | --- | --- |
| Fatty Liver | 1.99E-4 | DEPTOR, ACLY, ACOT13, G0S2, CCDC80, NR3C1, LECT2, PTGS2, FABP1, LPIN1, FASN, IGF1, IGFBP1, LMNA, GZMB, EBP, SIK2, NOCT, MAP2K1, PLTP, SOAT2, TPH1 |
| Steatohepatitis | 1.99E-4 | DEPTOR, RNF19A, ACLY, ACOT13, G0S2, CCDC80, NR3C1, LECT2, TLR8, PTGS2, DDIT3, FABP1, LPIN1, FASN, IGF1, IGFBP1, LMNA, CLU, GZMB, EBP, SIK2, DIO3, NOCT, MAP2K1, SOAT2, TPH1 |
| Glomerulosclerosis (disorder) | 2.87E-4 | RNF19A, TNC, RAPGEF5, ACLY, APOL1, PTGS2, DDIT3, CRY1, FASN, IGFBP1, CLU |
| Adult Fibrosarcoma | 2.87E-4 | RNF19A, TNC, ANXA3, NR3C1, COLQ, PTGS2, DDIT3, ARL4C, IGF1, CLU, EBP, MAFB, PLAU, ELK1, MAP2K1 |
| Fibrosarcoma | 3.97E-4 | RNF19A, TNC, ANXA3, NR3C1, COLQ, PTGS2, DDIT3, ARL4C, IGF1, CLU, EBP, MAFB, PLAU, ELK1, MAP2K1 |
| Lupus Erythematosus, Systemic | 7.13E-4 | NAT1, DDIT4, TIMD4, RNF19A, MFHAS1, RAPGEF5, STK17A, ANXA3, BACH2, PELI1, NR3C1, APOL1, TLR8, ZAP70, AR, PTGS2, GADD45A, LGALS3BP, IGF1, LMNA, KLF13, CLU, IL1R2, GZMB, EHF, FEN1, FOXO4, HES1, ELK1, MAP2K1, ALOX5AP, SOAT1 |
| Arteriosclerosis | 1.05E-3 | SOX9, RNF19A, TNC, RAPGEF5, ACLY, CCDC80, NR3C1, APOL1, LECT2, TLR8, AR, PTGS2, GADD45A, DDIT3, LGALS3BP, CRY1, IGF1, SERPINB9, IGFBP1, LMNA, CLU, PDGFD, GZMB, PACS2, FOXO4, MAFB, HES1, PLAU, ELK1, MAP2K1, PLTP, ALOX5AP, SOAT2, SOAT1, TPH1 |
| Dyslipidemias | 1.25E-3 | TIMD4, RAPGEF5, ACLY, ACOT13, NR3C1, LECT2, AR, PTGS2, FABP1, FASN, LMNA, MAFB, MAP2K1, PLTP, SOAT2 |
| Coronary Arteriosclerosis | 1.37E-3 | DDIT4, RNF19A, TNC, BACH2, NR3C1, APOL1, TLR8, AR, PTGS2, LGALS3BP, IGF1, SERPINB9, IGFBP1, LMNA, CLU, PDGFD, GZMB, EBP, FEN1, MAFB, HES1, PLAU, PLTP, ALOX5AP, SOAT2, SOAT1 |
| Adverse reaction to drug | 1.37E-3 | NAT1, NR3C1, TF, PTGS2, IGF1, IGFBP1, CLU |
| Drug toxicity | 1.37E-3 | NAT1, NR3C1, TF, PTGS2, IGF1, IGFBP1, CLU |
| Hyperinsulinism | 1.89E-3 | SOX9, KLF11, ACLY, NR3C1, LECT2, AR, DDIT3, LPIN1, FASN, IGF1, IGFBP1, LMNA, HES1, PLAU, PLTP |
| Hepatocarcinogenesis | 2.40E-3 | SOX9, RNF19A, LECT2, AR, PTGS2, LGALS3BP, IGDCC4, CCND2, CRY1, LPIN1, CKS1B, FASN, IGF1, IGFBP1, CLU, DIO3, HES1, PLAU, MAP2K1, SOAT1 |
| Coronary Artery Disease | 2.40E-3 | ERCC5, RNF19A, TNC, BACH2, NR3C1, APOL1, TLR8, ZAP70, AR, PTGS2, DDIT3, LGALS3BP, IGF1, IGFBP1, LMNA, CLU, IL1R2, PDGFD, GZMB, EBP, FEN1, MAFB, PLAU, CDH4, PLTP, ALOX5AP, SOAT2, SOAT1 |

**Table S6. Significantly enriched (FDR q value < 0.05) Gene Ontology (GO) Biological Processes (BP) in differentially expressed proteins (p < 0.05) in liver of Atlantic cod exposed to low and high dose WY-14,643.**

| GO BP | Protein symbol |
| --- | --- |
| small molecule biosynthetic process | ACACA, ACACB, ACLY, TECR, ASNS, ASPA, AACS, GLUL, NFYB, SYK,  APOA1, SAMHD1, APOE, NUDT9, ENOPH1, ALOX5AP |
| cholesterol biosynthetic process | ACACA, ACACB, ACLY, NFYB, APOA1, APOE |
| secondary alcohol biosynthetic process | ACACA, ACACB, ACLY, NFYB, APOA1, APOE |
| sterol biosynthetic process | ACACA, ACACB, ACLY, NFYB, APOA1, APOE |
| carboxylic acid biosynthetic process | ACACA, ACACB, ACLY, TECR, ASNS, ASPA, GLUL, SYK, ENOPH1,  ALOX5AP |
| organic acid biosynthetic process | ACACA, ACACB, ACLY, TECR, ASNS, ASPA, GLUL, SYK, ENOPH1,  ALOX5AP |
| fatty acid derivative biosynthetic process | ACACA, ACLY, TECR, AACS, SYK, ALOX5AP |
| fatty acid transport | CROT, ACACA, ACACB, SYK, APOE, FABP1 |
| monocarboxylic acid transport | CROT, ACACA, ACACB, SYK, APOE, SLC38A3, FABP1 |
| response to extracellular stimulus | ACACB, ASNS, AACS, GLUL, AIF1, LRP6, APOA1, APOE, TMBIM6,  SLC38A3, PCNA |
| long-chain fatty acid transport | ACACA, ACACB, SYK, APOE, FABP1 |
| regulation of platelet activation | VPS33B, SYK, APOE, DMTN |
| carboxylic acid metabolic process | GGH, CROT, ACACA, ACACB, ACLY, TECR, ASNS, ASPA, AACS,  GLUL, SYK, C3, ENOPH1, ALOX5AP, FABP1 |
| steroid biosynthetic process | ACACA, ACACB, ACLY, TECR, NFYB, APOA1, APOE |
| malonyl-CoA biosynthetic process | ACACA, ACACB |
| fatty acid metabolic process | CROT, ACACA, ACACB, ACLY, TECR, AACS, C3, ALOX5AP, FABP1 |
| immune effector process | GGH, IRF9, SERPINB9, TXNDC5, ISG15, ACLY, AGPAT2, SYK,  DNAJC5, C3, APOA1, IGLC1, SAMHD1, PRKDC, TMBIM6, IGKV4-1 |
| response to nutrient levels | ACACB, ASNS, AACS, GLUL, LRP6, APOA1, APOE, TMBIM6,  SLC38A3, PCNA |
| oxoacid metabolic process | GGH, CROT, ACACA, ACACB, ACLY, TECR, ASNS, ASPA, AACS,  GLUL, SYK, C3, ENOPH1, ALOX5AP, FABP1 |
| cholesterol metabolic process | ACACA, ACACB, ACLY, NFYB, APOA1, APOE |

**Table S7. Significantly enriched (FDR q value < 0.05) GO BP in differentially expressed proteins (p < 0.05) in liver of Atlantic cod exposed to GW501516.**

| GO BP Term | Protein symbol |
| --- | --- |
| SRP-dependent cotranslational protein targeting to membrane (GO:0006614) | RPL4, RPS15, RPS17, RPS29, RPL34, RPL27A, RPL36, RPL14,  RPL13, RPL38, RPS3A |
| cotranslational protein targeting to membrane (GO:0006613) | RPL4, RPS15, RPS17, RPS29, RPL34, RPL27A, RPL36, RPL14,  RPL13, RPL38, RPS3A |
| protein targeting to ER (GO:0045047) | RPL4, RPS15, RPS17, RPS29, RPL34, RPL27A, RPL36, RPL14,  RPL13, RPL38, RPS3A |
| viral gene expression (GO:0019080) | RPL4, RPS15, RPS17, RPS29, RPL34, RPL27A, RPL36, RPL14,  RPL13, RPL38, RPS3A |
| nuclear-transcribed mRNA catabolic process, nonsense-mediated decay (GO:0000184) | RPL4, RPS15, RPS17, RPS29, RPL34, RPL27A, RPL36, RPL14,  RPL13, RPL38, RPS3A |
| viral transcription (GO:0019083) | RPL4, RPS15, RPS17, RPS29, RPL34, RPL27A, RPL36, RPL14,  RPL13, RPL38, RPS3A |
| cellular protein metabolic process (GO:0044267) | RPL4, RPL34, FBXO2, SERPINA10, APOA1, RPS3A, RPS15,  RPS17, LGALS1, RPS29, RPL27A, RPL36, MXRA8, RPL14,  RPL13, RPL38, APOB |
| rRNA metabolic process (GO:0016072) | RPL4, RPS15, RPS17, RPS29, RPL34, RPL27A, RPL36, RPL14,  NOL8, RPL13, RPL38, RPS3A |
| rRNA processing (GO:0006364) | RPL4, RPS15, RPS17, RPS29, RPL34, RPL27A, RPL36, RPL14,  NOL8, RPL13, RPL38, RPS3A |
| peptide biosynthetic process (GO:0043043) | RPL4, RPS15, RPS17, RPS29, RPL34, RPL27A, RPL36, RPL14,  RPL13, RPL38, RPS3A |
| nuclear-transcribed mRNA catabolic process (GO:0000956) | RPL4, RPS15, RPS17, RPS29, RPL34, RPL27A, RPL36, RPL14,  RPL13, RPL38, RPS3A |
| ribosome biogenesis (GO:0042254) | RPL4, RPS15, RPS17, RPS29, RPL34, RPL27A, RPL36, RPL14,  NOL8, RPL13, RPL38, RPS3A |
| ncRNA processing (GO:0034470) | RPL4, RPS15, RPS17, RPS29, RPL34, RPL27A, RPL36, RPL14,  NOL8, RPL13, RPL38, RPS3A |
| viral process (GO:0016032) | RPL4, RPS15, RPS17, RPS29, RPL34, RPL27A, RPL36, RPL14,  RPL13, RPL38, RPS3A |
| translation (GO:0006412) | RPL4, RPS15, RPS17, RPS29, RPL34, RPL27A, RPL36, RPL14,  RPL13, RPL38, RPS3A |
| cytoplasmic translation (GO:0002181) | RPL4, RPS29, RPL27A, RPL36, RPL38, RPS3A |
| cellular macromolecule biosynthetic process (GO:0034645) | RPL4, RPS15, RPS17, RPS29, RPL34, RPL27A, RPL36, RPL14,  RPL13, RPL38, RPS3A |
| gene expression (GO:0010467) | RPL4, RPS15, RPS17, RPS29, RPL34, RPL27A, RPL36, RPL14,  RPL13, RPL38, RPS3A |
| triglyceride catabolic process (GO:0019433) | FABP1, FABP7, APOA1 |

**References:**

Perez-Silva, J. G., M. Araujo-Voces and V. Quesada (2018). "nVenn: generalized, quasi-proportional Venn and Euler diagrams." Bioinformatics **34**(13): 2322-2324.
